# T Lymphocytes from Chronic HCV-Infected Patients Are Primed for Activation-Induced Apoptosis and Express Unique Pro-Apoptotic Gene Signature

**DOI:** 10.1371/journal.pone.0077008

**Published:** 2013-10-10

**Authors:** Bin-Bin Zhao, Su-Jun Zheng, Lu-Lu Gong, Yu Wang, Cai-Feng Chen, Wen-Jing Jin, Ding Zhang, Xiao-Hui Yuan, Jian Guo, Zhong-Ping Duan, You-Wen He

**Affiliations:** 1 Key Laboratory of Systems Biology of Pathogens, Ministry of Health, Institute of Pathogen Biology, Chinese Academy of Medical Sciences & Peking Union Medical College, Beijing, R. P. China; 2 Beijing YouAn Hospital, Capital Medical University, Beijing, R. P. China; 3 Department of Immunology, Duke University Medical Center, Durham, North Carolina, United States of America; The University of Hong Kong, Hong Kong

## Abstract

Although extensive studies have demonstrated the functional impairment of antigen-specific CD4^+^ and CD8^+^ T-cells during chronic hepatitis C virus (HCV) infection, the functional status of global CD4^+^ and CD8^+^ T-cells remains unclear. In this report, we recruited 42 long-term (~20 years) treatment-naïve chronic HCV (CHC) patients and 15 healthy donors (HDs) to investigate differences in global CD4^+^ and CD8^+^ T-cells function. We show that CD4^+^ and CD8^+^ T-cells from CHC patients underwent increased apoptosis after TCR stimulation. Furthermore, IFN-γ, IL-9 and IP-10 were elevated in CHC patients’ plasma and promoted activation-induced T-cells death. Global CD4^+^ and CD8^+^ T-cells also showed unique transcriptional profiles in the expression of apoptosis-related genes. We identified BCL2, PMAIP1, and CASP1 in CD4^+^ T-cells and IER3 and BCL2A1 in CD8^+^ T-cells from CHC patients as HCV-specific gene signatures. Importantly, the gene expression patterns of CD4^+^ and CD8^+^ T-cells from CHC patients differ from those in CD4^+^ and CD8^+^ T-cells from human immunodeficiency virus type 1 (HIV-1) or hepatitis B virus (HBV) infected individuals. Our results indicate that chronic HCV infection causes a systemic change in cytokine levels that primes T-cells for activation-induced apoptosis. Furthermore, HCV infection programs unique apoptosis-related gene expression profiles in CD4^+^ and CD8^+^ T-cells, leading to their enhanced activation-induced apoptosis. These results provide novel insights to the pathogenesis of chronic HCV infection.

## Introduction

Approximately 170 million people are chronically infected with hepatitis C virus (HCV) [1,2]. The functional exhaustion of HCV-specific T-cells contributes to failed viral clearance in chronically infected patients [3–5]. HCV-specific CD8^+^ T-cells exhibit various degrees of functional impairment, including impaired proliferation, reduced effector cytokine production, and enhanced apoptosis [6–8]; however, the mechanisms underlying these changes are incompletely understood. Deregulated expression of inhibitory receptors on HCV-specific CD8^+^ T-cells, increased Treg numbers, and enhanced cytokine levels impact the function of antigen-specific T-cells during chronic HCV infection [3,4]. Nevertheless, the impact of chronic HCV infection on the function of global CD4^+^ and CD8^+^ T lymphocytes remains poorly understood. Addressing this issue will elucidate mechanisms by which the host environment impacts antigen-specific T-cells during chronic HCV infection. 

In this study, we investigated the functional status of global CD4^+^ and CD8^+^ T-cells in a group of long-term (~20 years) treatment-naive chronic HCV infected (CHC) patients. We found that long-term chronic HCV infection did not cause a significant change in the numbers of total lymphocytes or major lymphocyte subpopulations or in total T-cells proliferation. In contrast, both CD4^+^ and CD8^+^ T-cells from CHC patients exhibited dramatically enhanced activation-induced apoptosis. Furthermore, IFN-γ, IP-10, and IL-9 were significantly elevated in the plasma of CHC patients. These cytokines sensitized primary T-cells from healthy donors (HDs) to activation-induced death. Consistent with these findings, CD4^+^ and CD8^+^ T-cells from CHC patients displayed deregulated expression of apoptosis-related genes. B-cell lymphoma 2 (BCL2), phorbol-12-myristate-13-acetate-induced protein 1 (PMAIP1), and caspase 1 (CASP1) in CD4^+^ T-cells and immediate early response 3 (IER3) and BCL2-related protein A1 (BCL2A1) in CD8^+^ T-cells from CHC patients are identified as HCV-specific gene signatures. These gene signatures were specific to CHC and differed from those in T-cells from chronic human immunodeficiency virus type 1 (HIV-1) or hepatitis B virus (HBV) patients. Taken together, our results demonstrate that chronic HCV infection induces a cytokine environment that sensitizes T-cells to activation-induced apoptosis and induces unique apoptosis-related gene signatures. 

## Materials and Methods

### Subjects

Forty-two anti-HCV Ab^+^ CHC patients without prior anti-viral treatment from Gansu province, China were recruited. The CHC patients were infected with HCV through blood donation during the 1980s and 1990s (1988-1994). The average time of infection was ~20 years. Fifteen HDs in the same age range were selected as healthy controls. The clinical parameters were measured in Beijing YouAn Hospital (Table **1**). Written informed consent was obtained from all individuals before their participation in this study, and the study protocol was approved by Beijing YouAn Hospital Ethical Committee. 

**Table 1:** Clinical parameters of CHC patients and HDs.

**Parameter**	**CHC Patients**	**Healthy Donors**
Number in each group	42	15
Age (years), median (range)	51.6 (35-67)	44.5 (37-56)
Sex (M:F)	25:17	1:4
Serum HCV RNA (IU/ml) (range)	0-6.38×10^7^	---
Serum HCV RNA gene type	1b, 1b/2a, 2a, 2a/2b (16:1:19:1)	---
Serum total bilirubin (μmol/L), median (range)	15.1 (6.9-36.7)	8.37 (6.1-12.8)
Serum direct bilirubin (μmol/L), median (range)	3.1 (1.7-6.4 )	2.02 (0.4-4.3)
AST (IU/L), median (range)	51.2 (18.4-389.0)	48.45 (26.8-81.9)
ALT (IU/L), median (range)	63.0 (10.6 - 590.8)	16.2 (8.6-44.3)

### Flow cytometry

Venous blood samples were collected using EDTA as an anticoagulant. Red blood cells were lysed with Lysing Buffer (BD Biosciences, CA, USA), and the cells were stained with APC-anti-CD4 (RPA-T4), PerCP-anti-CD8 (SK1) (BD Biosciences), FITC-anti-CD3 (OKT3), FITC-anti-CD45RO (HI100), and PE-anti-CD45RA (UCHL1) (Biolegend, CA, USA) following the manufacturers’ instructions. Flow cytometry data were collected with a BD FACSAria™ Cell Sorter (BD Biosciences) and analyzed with FlowJo (Tree Star, OR, USA).

### PBMC separation and T-cells stimulation

Peripheral blood mononuclear cells (PBMCs) were isolated from fresh blood samples by density gradient centrifugation using Ficoll-Paque PLUS (GE Healthcare, Buckinghamshire, UK). CD4^+^ and CD8^+^ T-cells were purified from PBMCs using CD4 and CD8 microbeads with an autoMACS Separator (Miltenyi Biotec, CA, USA) following the manufacturer’s instructions. The purity of separated CD4^+^ and CD8^+^ T-cells was >90%. For T-cells stimulation assays, 96-well plates (Costar Corning, MA, USA) were coated overnight at 4°C or for 2-3 hours at 37°C with 1 μg/ml anti-CD3 and anti-CD28 (eBioscience, CA, USA). PBMCs were labeled with 1 μl 5-(and 6-)-carboxyfluorescein diacetate, succinimidyl ester (CFSE, 5mM, Sigma-Aldrich, MO, USA) in a volume of 1 ml PBS and seeded at 1-1.5×10^6^ cells/ml in the coated 96-well plates for 3 days. T-cells proliferation was measured by CFSE dilution, and T-cells apoptosis was determined using Pacific Blue Annexin V (Biolegend, CA, USA). PBMCs without stimulus were cultured for 3 days to determine the spontaneous apoptosis of T-cells.

### Cytokine measurement and sensitization culture

Plasma samples were collected by centrifuging fresh blood samples at 400×g for 5 minutes. Twenty-seven cytokines, including Eotaxin, FGF basic, G-CSF, GF-CSF, IFN-γ, IL-1β, IL-1Ra, IL-2, IL-4, IL-5, IL-6, IL-7, IL-8, IL-9, IL-10, IL-12 (p70), IL-13, IL-15, IL-17, IP-10, MCP-1 (MCAF), MIP-1a, MIP-1b, PDGF bb, RANTES, TNF-α and VEGF, were measured with a human Grp I Cytokine 27-Plex Panel (Bio-Rad, CA, USA) using a Luminex 200 (Millipore, MA, USA) according to the manufacturer’s instructions. To test the effects of cytokines on T-cells apoptosis and proliferation, freshly isolated CFSE-labeled PBMCs from HDs were cultured in RPMI 1640 medium (10% FBS and 7.5pg/ml IL-7) containing cytokines at the concentrations identified in the plasma of CHC patients (IFN-γ 300, 500pg/ml; IL-1β 12pg/ml; IL-9 60, 400pg/ml; IP-10 7000pg/ml) (Peprotech, NJ, USA). PBMCs were incubated at 37°C in the presence of 5% CO_2_ in 96-well plates for 48 hours and then transferred to plates coated with anti-CD3 and anti-CD28 antibodies for stimulation. PBMCs were cultured without cytokines as controls (NC) in the same condition. T-cells apoptosis and proliferation were measured after 3 days of stimulation.

### Microarray analysis

Purified CD4^+^ and CD8^+^ T-cells were lysed in Trizol (Invitrogen, CA, USA), and RNA extraction and microarray assays were performed by CapitalBio Corporation (Beijing, China). The quality and integrity of total RNA were determined using an Agilent 2100 Bioanalyzer (Agilent Technologies, CA, USA). T-cells gene expression patterns were detected with an Affymetrix GeneChip® Human Genome U133 Plus 2.0 Array (Affymetrix, CA, USA). Five patients and 5 HDs randomly selected from sample groups (HCV-h group, HCV-l group, and HD group) were analyzed for each cell type. 

### Statistical analysis

CEL files were imported to Partek Genomics Suite software (Partek Inc., MO, USA) as raw data using default settings. After background correction, normalization, and summarization using RMA (Robust Multichip Average) analysis, expression data were log_2_ transformed for further analysis. Differential gene expression between CHC patients and HDs was determined by one way ANOVA. False discovery rate (FDR) correction was applied using the step-up method with an FDR of 5% as the cut-off. Significantly modulated genes were defined as those with *P* <0.05 and ≥1.5-fold change in mean differential expression. Significantly modulated apoptosis genes were hierarchically clustered using Partek Genomics Suite software with default settings. Additionally, principal components analysis (PCA) was used to cluster patients in 3 dimensions. The significantly changed genes were used as input for the gene function and pathway enrichment analysis. GeneGo Metacore software (GeneGO Inc., CA, USA) was used to identify the most significant biological pathways associated with the modulated genes using default parameters. Different HCV patients groups (HCV-h or HCV-l) were performed separately and compared to each other for common pathways. In addition, common significantly modulated genes shared by both HCV patient groups were imported for pathway analysis. DAVID Bioinformatics Resources (http://david.abcc.ncifcrf.gov/) was used to identify functional categories based on the annotation sources GOTERM-BP (biological process). Different HCV patient groups were performed separately and the FDR for enrichment analysis was set as 5%. Significantly modulated genes were classified into different functional categories according to the enrichment results, as listed in Table S1 and S2. Microarray data for gene expression in global CD4^+^ and CD8^+^ T-cells during chronic HBV [9] and HIV-1 infection [10,11] were obtained from public databases Gene Expression Omnibus (GEO). The same statistical analysis for HBV and HIV-1 data was done as described above. The common significantly modulated genes between chronic HCV infection and chronic HIV-1 or HBV infection were identified by gene symbols, which were also analyzed for pathway and function enrichment. For comparison of special T-cells gene expression signature, log_2_ values were used and statistic strategy for quantitative real-time PCR was applied. For correlation analysis of apoptosis gene expression level with clinical parameters or plasma levels of cytokines, fluorescent values of all samples from microarray analysis instead of log_2_ values were used.

GraghPad Prism 5.00 software was applied for data analysis except in microarray analysis, and p-values<0.05 (t-test) were regarded as statistically significant. Microarray raw data are available in the Gene Expression Omnibus database (http://www.ncbi.nlm.nih.gov/geo) under accession no.GSE49954.

## Results

### CHC patient characteristics

**Figure 1:**
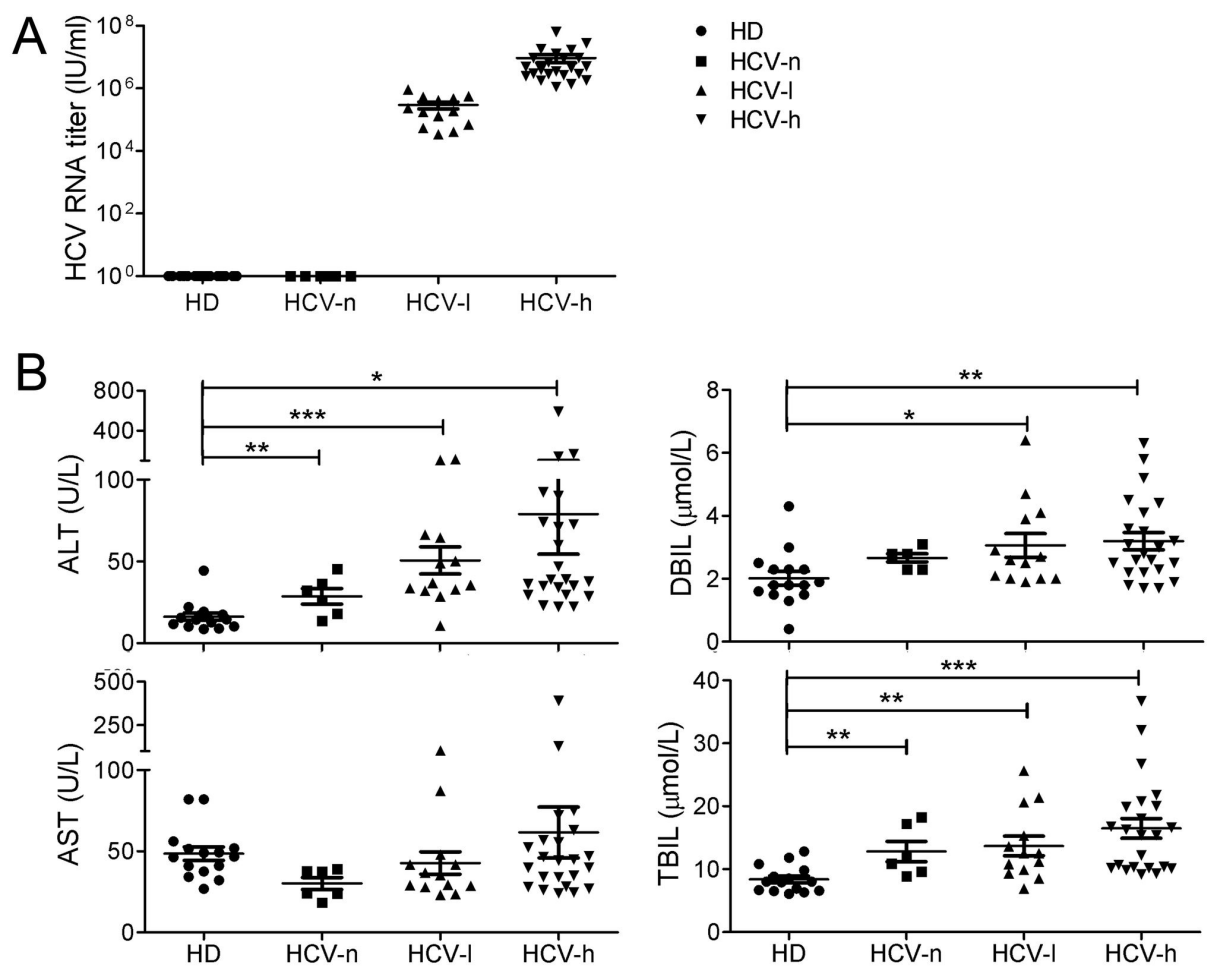
Clinical parameters of the three groups of CHC patients (HCV-n group, n=6; HCV-l group, n=13; HCV-h group, n=23) and HDs. (A), HCV RNA titers for the three groups of CHC patients. HCV-n: negative; HCV-l:10^3^-10^6^ IU/ml; HCV-h: ≥10^6^ IU/ml. (B), The concentrations of ALT, AST, DBIL, and TBIL in the plasma of CHC patients compared to HDs. *, p<0.05; **, p<0.01; ***, p<0.001.

Viral load and CTL function are inversely correlated during HIV and HBV infection [12–15]. To determine the impact of serum HCV titers on the function of global CD4^+^ and CD8^+^ T-cells in CHC patients, we divided the CHC patients into 3 groups: HCV-n (negative), HCV-l (low) (10^4^-10^6^ IU/ml RNA), and HCV-h (high) (10^6^-10^8^ IU/ml RNA) (Figure **1A**). Alanine transaminase (ALT) and total bilirubin (TBIL) values were significantly higher in all 3 groups of CHC patients than in HDs (Figure **1B**). Although the direct bilirubin (DBIL) values in HCV-l and HCV-h patients were within the normal range, they were significantly higher than those in HDs (Figure **1B**). Consistent with previous reports, 12% of the CHC patients exhibited abnormal liver function based on TBIL and ALT levels [16]. Six large groups of viral genotypes (1-6) containing over 70 different subtypes are defined based on subgenomic regions [17]. In China, the main subtypes are 1b and 2a (Table **1**). There was no correlation between HCV genotypes and RNA titers (data not shown). Although the mean ALT, AST, DBIL, and TBIL values in the HCV-h group were higher than those in the other groups (Figure **1B**), we did not find a significant correlation between HCV viral titers and gender, age or ALT, AST, DBIL, and TBIL values in these long-term treatment-naive patients (Figure **S1**). 

### Enhanced activation-induced apoptosis of T-cells from CHC patients

**Figure 2:**
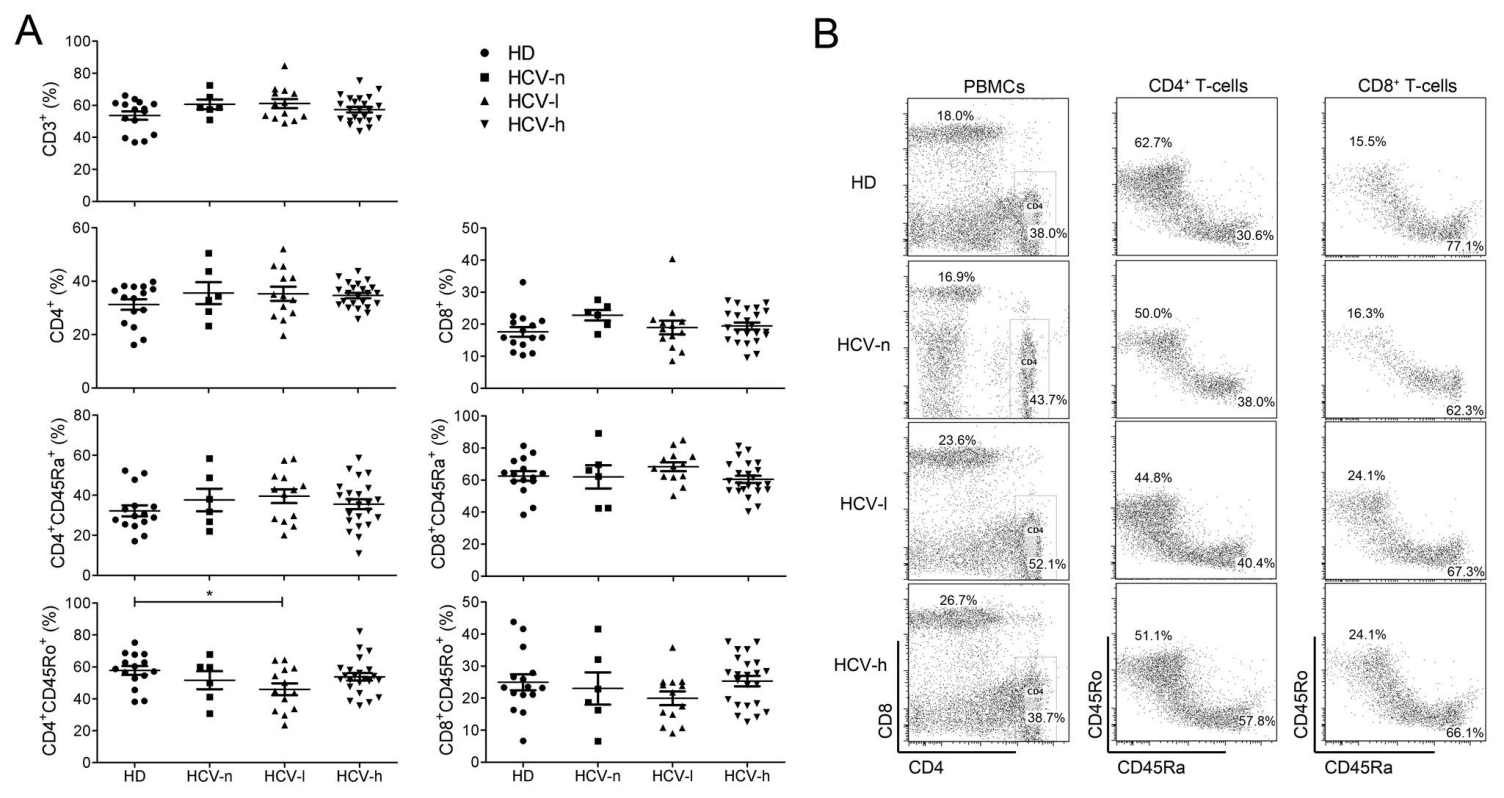
Percentages of T-cells subpopulations in the peripheral blood of CHC patients and HDs. (A), The percentages of total T-cells (CD3^+^), CD8^+^, and CD4^+^ T-cells in the peripheral blood of patients and HDs. The frequency of memory/activated (CD45RO^+^) T-cells and naïve (CD45RA^+^) T-cells among CD8^+^ or CD4^+^ T-cells was also determined. (B), Representative FACS profiles of T-cells subsets in the peripheral blood of patients and HDs are shown. *, p<0.05.

Although HCV-specific T-cells have been thoroughly studied in CHC patients [3], the functional status of total T-cells in CHC patients is not clear. We first assessed the frequencies of lymphocyte subpopulations in CHC patients. Interestingly, the frequencies of most of the T-cells subpopulations in the peripheral blood of CHC patients were similar to those in HDs (Figure **2A and 2B**). The only significant change was a reduction in CD4^+^CD45RO^+^ T-cells in HCV-l patients (HD, 57.8% ± 2.8%; HCV-l, 46.0% ± 3.8%) (Figure **2A**), suggesting that long-term chronic HCV infection had a minimal impact on overall lymphocyte composition. 

**Figure 3:**
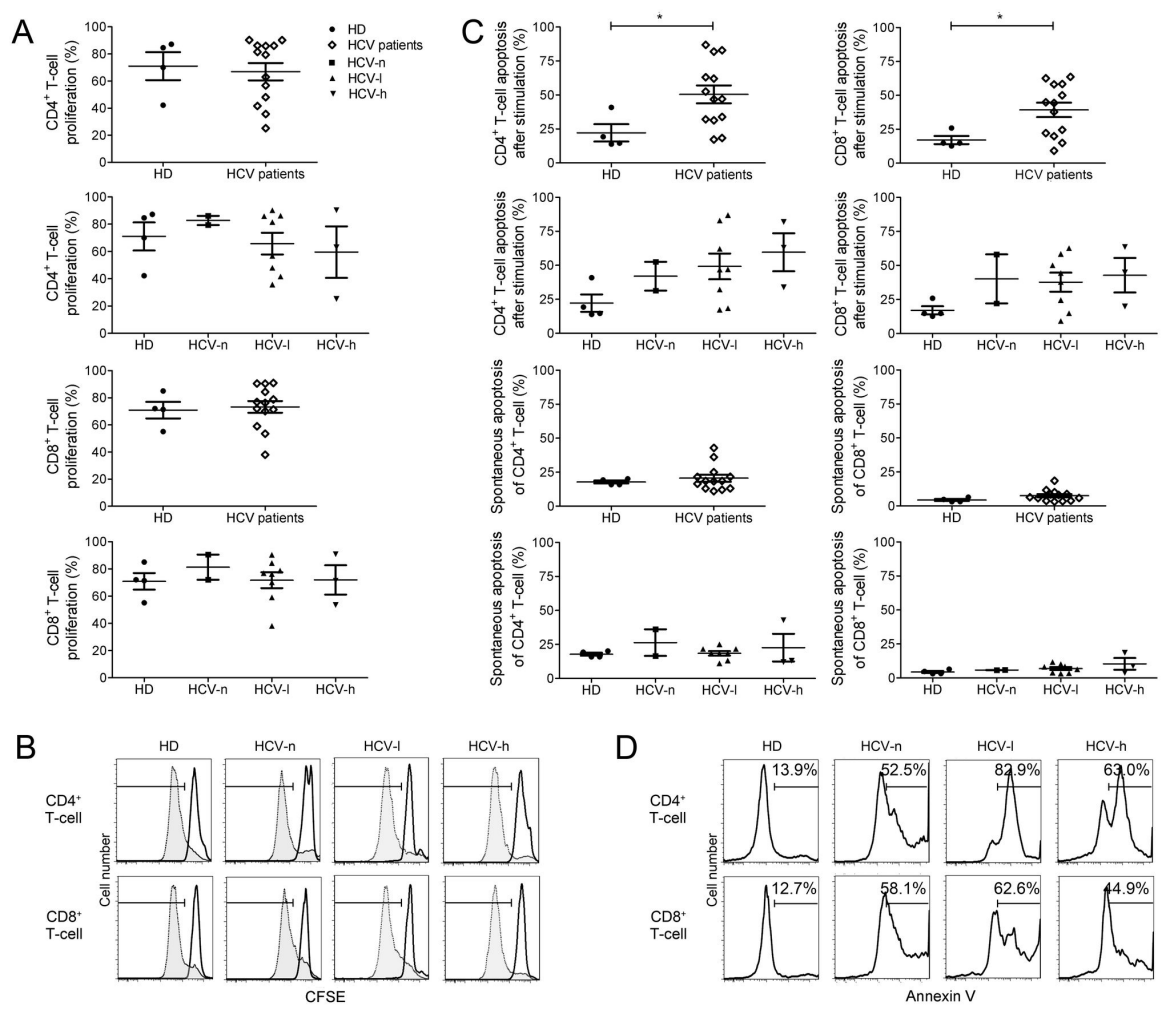
Enhanced activation-induced apoptosis of global CD4^+^ and CD8^+^ T-cells from CHC patients. Freshly isolated PBMCs were stimulated with plate-bound anti-CD3/CD28 for 3 days. T-cells proliferation was measured by CFSE dilution, and apoptosis was measured by Annexin V staining. (A), Proliferation rates of total CD4^+^ and CD8^+^ T-cells from the CHC and HD groups. (B), Representative FACS profiles of T-cells proliferation for each group of CHC patients and HDs. (C), Apoptosis rates of CD4^+^ and CD8^+^ T-cells from the CHC patients and HDs with or without anti-CD3/CD28 stimulation. (D), Representative FACS profiles of T-cells apoptosis for each group of CHC patients and HDs. For (A) and (C), each dot represents one individual. *, p<0.05.

We next examined the proliferation and apoptosis of total CD4^+^ and CD8^+^ T-cells from CHC patients. Surprisingly, we did not observe any significant difference in the proliferation of total CD4^+^ or CD8^+^ T-cells from CHC patients and HDs (Figure 3A and 3B). However, total T-cells from all CHC groups exhibited significantly enhanced activation-induced apoptosis, with a >2-fold increase in CD8^+^ T-cells and a ~2-3-fold increase in CD4^+^ T-cells when compared to T-cells from HDs (Figure 3C and 3D). In contrast, the spontaneous apoptosis rates of total CD4^+^ and CD8^+^ T-cells were similar among the CHC patients and HDs (Figure **3C**). These results suggest that changes in the host environment induced by HCV infection may sensitize the T-cells to activation-induced apoptosis. 

### The roles of cytokines in activation-induced apoptosis of T-cells from CHC patients

**Figure 4:**
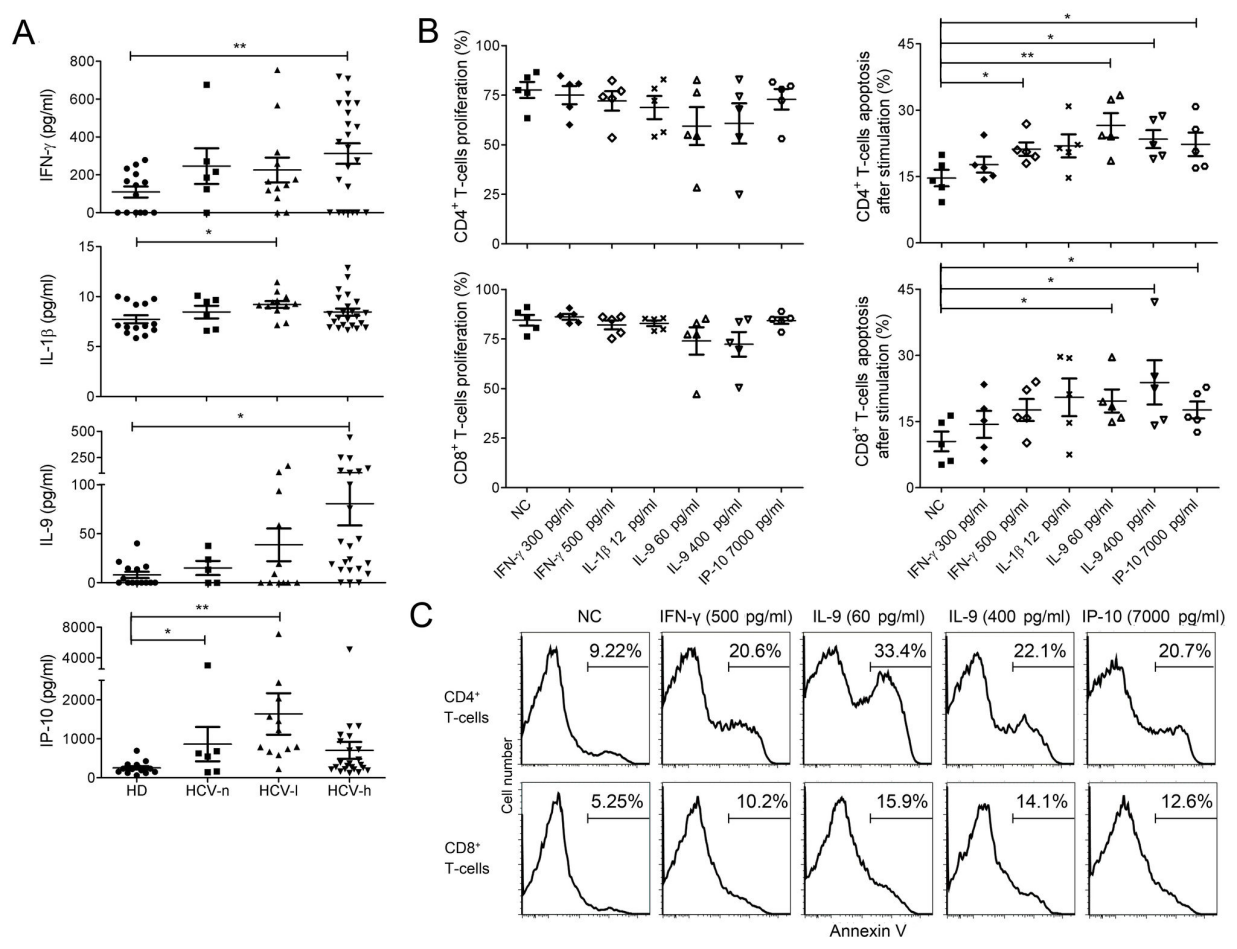
Elevated cytokine levels in the plasma of CHC patients and their roles in sensitizing T-cells to activation-induced apoptosis. (A), Cytokine levels, including IFN-γ, IL-1β, IL-9, and IP-10, in the plasma of CHC patients and HDs as measured by Luminex assay. (B), Effect of pre-incubation of PBMCs with the indicated cytokines on T-cells proliferation as measured by CFSE dilution and T-cells apoptosis as measured by Annexin-V staining. PBMCs from HDs were pre-incubated with or without (NC) the indicated cytokines for 48 hrs and then stimulated with anti-CD3/CD28 for 3 days before the measurement of proliferation and apoptosis. Data were from 5 independent HDs. (C), Representative FACS profiles of CD4^+^ and CD8^+^ T-cells apoptosis after pre-incubation with cytokines followed by stimulation. *, p<0.05; **, p<0.01.

Cytokines play important roles in regulating T-cells survival. We hypothesized that dysregulated cytokine expression in the CHC patients might sensitize CD4^+^ and CD8^+^ T-cells to activation-induced apoptosis. Plasma levels of IFN-γ, IL-1β, IL-9, and IP-10 were elevated in the CHC patients (Figure **4A**). Interestingly, the levels of IFN-γ and IL-9 were highest in the HCV-h group, whereas the levels of IL-1β and IP-10 were highest in the HCV-l group (Figure **4A**). The levels of other primary inflammatory cytokines, such as TNF-α, were similar in CHC patients and HDs (data not shown). 

We next examined whether these cytokines sensitized CD4^+^ and CD8^+^ T-cells to activation-induced cell death. PBMCs from HDs were pre-cultured with IFN-γ, IL-1β, IL-9, or IP-10 for 48 hours before stimulation with anti-CD3/CD28 antibodies. The concentrations of cytokines used were equivalent to either the median or the maximal value measured in the CHC patients. At these concentrations, these four cytokines did not significantly alter TCR-induced CD4^+^ and CD8^+^ T-cell proliferation (Figure **4B**), suggesting that the abnormal levels of these cytokines did not cause gene expression changes related to the cell proliferation machinery. In contrast, pre-incubation of HD T-cells with IFN-γ, IL-9, or IP-10 resulted in significantly enhanced activation-induced CD4^+^ or CD8^+^ T-cells apoptosis (Figure 4B and 4C); however, pre-incubation of T-cells with IL-1β did not affect activation-induced cell death (Figure **4B**) (p>0.05). These results demonstrate that IL-9, IFN-γ and IP-10 sensitize T-cells to activation-induced apoptosis and suggest that cytokine levels in CHC patients affect global T-cells function. 

### Gene expression profiles of CD4^+^ and CD8^+^ T-cells in CHC patients

**Figure 5:**
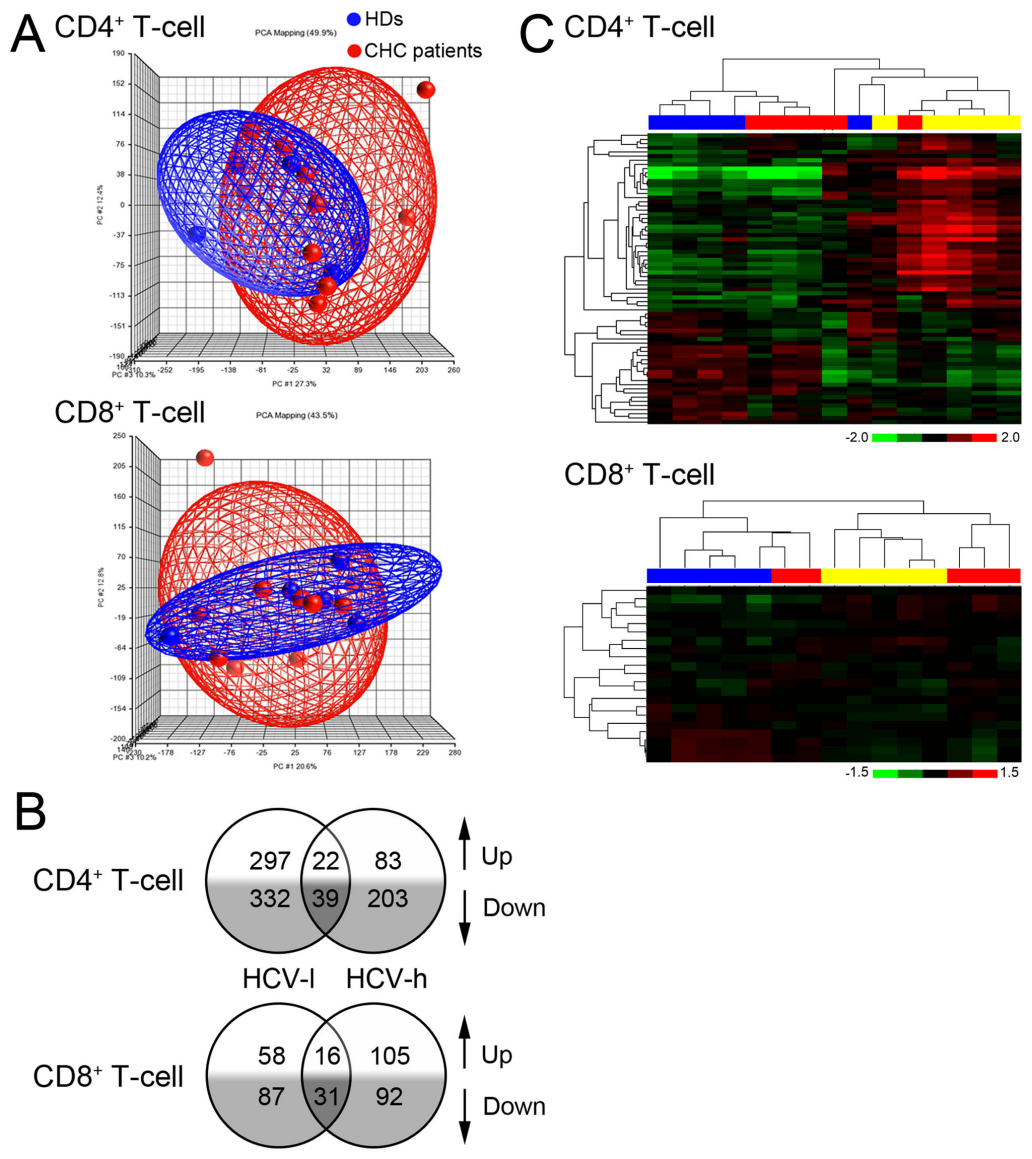
Overall gene expression profiles of CD4^+^ or CD8^+^ T-cells from CHC patients. (A), Scatterplot graph of CD4^+^ and CD8^+^ T-cells with PCA (principal component analysis) mapping. CHC patients, n=10; HDs, n=5. (B), The number of genes differentially expressed (fold-change ≥1.5, p<0.05) in CD4^+^ and CD8^+^ T-cells of HCV-l and HCV-h group when compared to those in HDs. The microarray data were derived from 5 individuals for each group. The numbers in the overlapping region represent the genes changed in both the HCV-l and HCV-h groups. (C), Clustering analysis of significantly changed apoptosis-related genes in CD4^+^ and CD8^+^ T-cells from CHC patients and HDs. Blue blocks represent HDs; yellow blocks represent HCV-l samples; red blocks represent HCV-h samples.

These results suggest that the host environment in CHC patients may reprogram the gene expression profiles of CD4^+^ and CD8^+^ T-cells. We thus sought to define the gene expression profiles of total CD4^+^ and CD8^+^ T-cells from CHC patients. Surprisingly, the overall gene expression patterns of global CD4^+^ or CD8^+^ T-cells from CHC patients were not dramatically different from those from HDs and cannot be used to separate CHC patients from the HDs (Figure **5A** and Figure **S2**). Interestingly, T-cells from HCV-h and HCV-l patients shared only a few common changed genes (61 genes for CD4^+^ T-cells and 47 genes for CD8^+^ T-cells) (Figure **5B**). Furthermore, no common pathway was altered in T-cells from both HCV-h and HCV-l patients (Figure **S3**). These results suggest that differences in HCV titers influence the gene expression patterns of CD4^+^ and CD8^+^ T-cells in CHC patients. 

We further analyzed the gene expression changes using the online DAVID program. Significantly changed genes involved in important biological processes for CD4^+^ and CD8^+^ T-cell function were selected from the HCV-h and HCV-l groups (Table **S1** and **S2**). Although the inhibitory receptors PD-1, Tim3, 2B4, and CTLA-4 are upregulated in HCV-specific CD8^+^ T-cells in CHC patients [18–24], the mRNA expression of these receptors was not significantly changed in global CD4^+^ or CD8^+^ T-cells of CHC patients, suggesting that the inhibitory receptors do not contribute to global T-cells dysfunction during chronic HCV infection.

Because global CD4^+^ and CD8^+^ T-cells from the CHC patients exhibited enhanced activation-induced apoptosis, we analyzed the expression profiles of genes related to apoptosis. The number of significantly changed apoptosis-related genes was greater in CD4^+^ T-cells from the CHC patients than in CD8^+^ T-cells (Table **S1** and **S2**). Interestingly, the significantly changed apoptosis-related genes in CD4^+^ T-cells were largely different from those in CD8^+^ T-cells (Table **S1** and **S2**), suggesting that two different sets of genes may be involved in the enhanced activation-induced apoptosis of CD4^+^ and CD8^+^ T-cells. Furthermore, genes altered in the HCV-l group were different from those in the HCV-h group for both CD4^+^ and CD8^+^ T-cells (Table **S1** and **S2**), suggesting that differences in HCV titers have a qualitative effect on T-cells gene expression profiles. Importantly, the expression signatures of the apoptosis-related genes in CD4^+^ and CD8^+^ T-cells of CHC patients were distinct from those of HDs and were sufficient to distinguish the CHC patients from HDs (Figure **5C**). These results demonstrate that chronic HCV infection results in altered expression of apoptosis-related genes in global CD4^+^ and CD8^+^ T-cells. 

### Correlation of apoptotic gene expression levels with the pathogenesis of HCV infection

**Figure 6:**
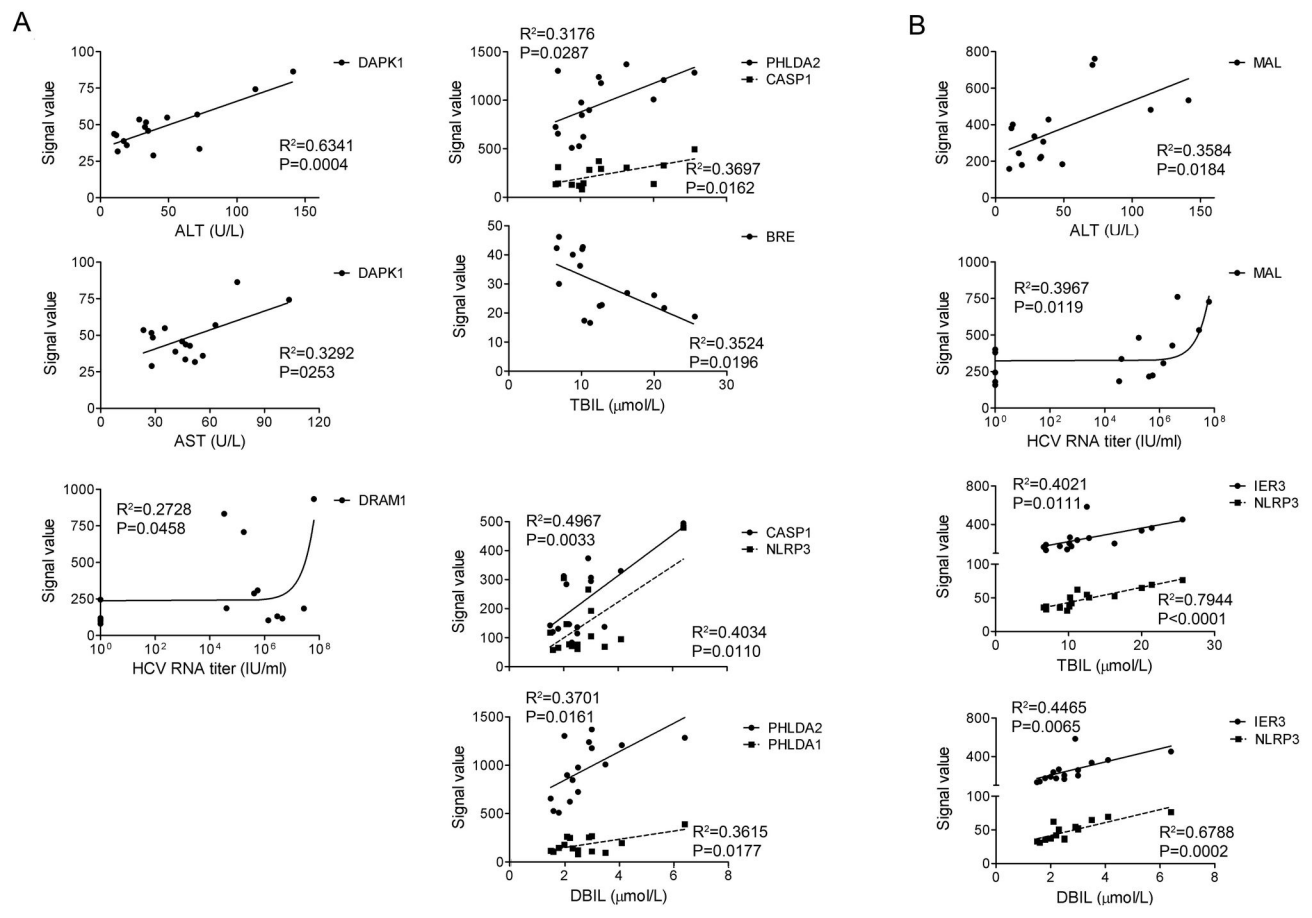
Correlation between the expression levels of apoptosis-related genes and clinical parameters in CHC patients. (A), Correlation of the expression levels of apoptosis-related genes in CD4^+^ T-cells from CHC patients with clinical parameters. (B), Correlation of the expression levels of the apoptosis-related genes in CD8^+^ T-cells from CHC patients with clinical parameters. The original fluorescent signal values for each gene detected in microarray assays were used for the correlation study.

We analyzed the significance of the expression levels of apoptotic genes during HCV infection by correlating them with other clinical parameters. We found that the expression levels of 20 apoptosis-related genes in CD4^+^ T-cells and 6 apoptosis-related genes in CD8^+^ T-cells were significantly correlated (p<0.05) with at least one clinical parameter (Figure **6** and data not shown). Interestingly, the expression levels of 5 apoptosis-related genes death-associated protein kinase 1 (DAPK1), pleckstrin homology-like domain family A member 1 (PHLDA1), pleckstrin homology-like domain family A member 2 (PHLDA2), CASP1, and NLR family pyrin domain-containing 3 (NLRP3) in CD4^+^ T-cells from CHC patients were positively correlated with ALT, AST, TBIL, and DBIL values (Figure **6A**). Expression of the anti-apoptotic gene brain and reproductive organ-expressed (BRE) was negatively correlated with TBIL. DNA damage-regulated autophagy modulator 1 (DRAM1) expression in CD4^+^ T-cells was correlated with HCV RNA titers in the CHC patients (Figure **6A**). Interestingly, the expression of NLRP3 in CD8^+^ T-cells from CHC patients was positively correlated with TBIL and DBIL values (Figure **6B**). In addition, the expression levels of mal, T-cell differentiation protein (MAL) in total CD8^+^ T-cells were positively correlated with the ALT values and HCV RNA titers (Figure **6B**). These significant correlations suggest that dysregulation of T-cells death may be associated with the pathogenesis of chronic HCV infection.

### Gene expression signatures of total CD4^+^ and CD8^+^ T-cells in chronic HCV infection differ from those in chronic HIV or HBV infection

**Figure 7:**
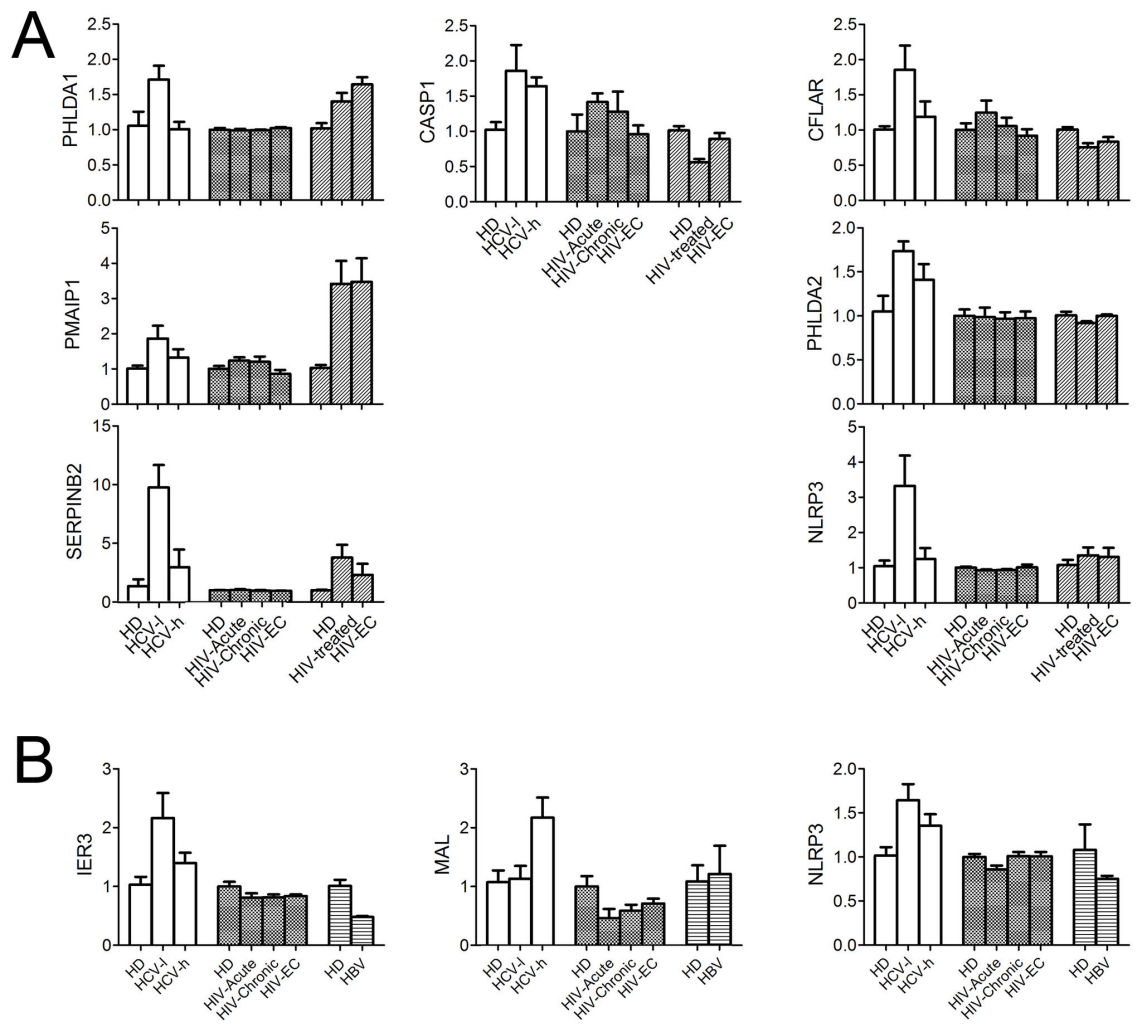
Expression patterns of apoptosis-related genes in T-cells from patients infected with HCV, HIV-1, and HBV. (A), Relative expression levels of apoptosis-related genes in CD4^+^ T-cells during HCV or HIV-1 infection. (B), Relative expression levels of apoptosis-related genes in CD8^+^ T-cells during HCV, HIV-1, or HBV infection. The gene expression levels in T-cells from HDs were defined as 1, and fold changes are shown.

An important feature of untreated HIV infection is the gradual loss of global CD4^+^ T-cells via apoptosis [25]. We compared the gene expression profiles of CD4^+^ and CD8^+^ T-cells from CHC patients to those of HIV or HBV patients using published microarray data [9–11]. Interestingly, the expression of 65 genes was significantly changed in both CHC and HIV patients (Table **S3**). Despite these commonly changed genes, the T-cells gene expression patterns in these two groups of patients differed greatly. First, the expression of interferon-stimulated genes (ISGs) including interferon alpha-inducible protein 6 (IFI6), interferon alpha-inducible protein 27 (IFI27), interferon-inducible protein 44 (IFI44), 2'-5'-oligoadenylate synthetase 1 (OAS1), and myxovirus resistance protein 1 (MX1), was upregulated early during the acute phase of HIV-1 infection and maintained through the chronic phase but was not upregulated in CD4^+^ T-cells from CHC patients. Second, many apoptosis-related gene expression changes in CD4^+^ T-cells from CHC patients were not observed in CD4^+^ T-cells from HIV-1 infected patients (Figure **7A** and Table **S3**). These results suggest that HCV and HIV infection have different impacts on the expression profiles of apoptosis-related genes in total CD4^+^ T-cells. 

Like chronic HCV infection, chronic HBV (CHB) infection also causes functional exhaustion of both antigen-specific and global CD8^+^ T-cells [13,15,26–28]. We previously identified gene changes in total CD8^+^ T-cells from CHB patients [9]. Only 15 genes were altered during both CHC and CHB infection, and 6 of these genes displayed opposite changes in CHC and CHB patients (Table **S4**). The ISG signature associated with CD8^+^ T-cells during acute and chronic HIV-1 infection [10,11] was not shared with CD8^+^ T-cells from CHC patients. Importantly, the expression levels of the majority of the apoptosis-related genes that were altered in CD8^+^ T-cells from CHC patients either were not changed or displayed opposite changes in CD8^+^ T-cells from patients chronically infected with HBV or HIV-1 (Figure **7B** and Table **S4**, Table **S5**). Taken together, these results demonstrate that global CD8^+^ T-cells express a unique apoptosis-related gene signature during chronic HCV infection that differs from that observed during chronic HBV or HIV-1 infection. 

## Discussion

Although the functional exhaustion of HCV-specific T-cells during CHC is well established, the functional status of total CD4^+^ and CD8^+^ T-cells in CHC patients remains poorly understood. In this study, we examined the functional status and gene expression profiles of total CD4^+^ and CD8^+^ T-cells in a group of long-term treatment-naïve CHC patients. Total CD4^+^ and CD8^+^ T-cells from CHC patients exhibited enhanced activation-induced apoptosis but proliferated normally. The plasma levels of IFN-γ, IL-9, and IP-10 in these patients were significantly elevated, and pre-incubation of primary T-cells from healthy donors with these cytokines sensitized T-cells to activation-induced apoptosis. Furthermore, although the overall gene expression pattern in CD4^+^ and CD8^+^ T-cells from CHC patients was indistinguishable from that from HDs, CHC patients’ T-cells expressed unique apoptosis-related gene signatures that differed from those in T-cells from patients chronically infected with HIV-1 or HBV. Our data provide important insight into the functional status of global T-cells during chronic HCV infection and suggest that the host environment during chronic HCV infection alters T-cells function. 

The enhanced activation-induced apoptosis of total CD4^+^ and CD8^+^ T-cells from CHC patients is likely due to the combined effects of several cytokines, including IFN-γ, IP-10, and IL-9. The role of IFN-γ in priming T-cells for activation-induced death has been firmly established [29,30]. Moreover, elevated IFN-γ in CHC patients modulates macrophage activation, and IFN-γ can directly act on T-cells or indirectly induce T-cells death by promoting the production of galectin-9 by monocytes and macrophages during chronic HCV infection [31]. Galectin-9 induces the apoptosis of HCV-specific CTLs by interacting with its cellular receptor, T cell immunoglobulin mucin-3 (Tim-3) [31]. Elevated IP-10 in CHC patients is a predictor of poor therapeutic outcome [32,33]. A previous report also showed that in combination with IL-2 and IFN-α, IP-10 induces the activation-independent apoptosis of primary human T-cells [34]. Our data clearly demonstrate that IP-10 also sensitizes primary human T-cells to activation-induced apoptosis. 

Surprisingly, we found that a high concentration of IL-9 promotes activation-induced apoptosis in T-cells. IL-9, a common γc-sharing cytokine, is a T-cells growth factor [35,36]. Neutralization or genetic deletion of IL-9 in mouse models dramatically reduced the effector T-cells population, suggesting a role for IL-9 in promoting T-cells survival and proliferation [37,38]. However, in our study, T-cells cultured in high concentrations of IL-9 were more sensitive to activation-induced apoptosis. Two possible mechanisms might account for this observation. First, high concentrations of IL-9 might induce an altered expression pattern of pro- versus anti-apoptotic genes in human T-cells. IL-4, another γc-sharing cytokine, was recently shown to induce the upregulation of the pro-apoptotic genes Bcl-2-like protein 11 (Bim) and Bcl-2-associated X (Bax) in T-cells [39]. Second, IL-9 may act indirectly by inducing pro-apoptotic cytokines in other PBMC populations. The precise mechanisms by which IL-9 primes human T-cells for activation-induced apoptosis require further investigation. Nevertheless, high plasma IL-9 levels are associated with poor treatment outcomes in HIV/HCV-coinfected patients [40] and in patients with chronic heart failure [41]. Together, these data suggest that high plasma IL-9 levels are pathogenic during chronic HCV infection. Importantly, our results strongly suggest that these cytokines act together on both global T-cells and HCV-specific T-cells. Thus, to restore the impaired survival of antigen-specific T-cells in CHC patients, it will be essential to neutralize the pro-apoptotic activities of these cytokines. 

 Although the overall gene expression patterns of CD4^+^ and CD8^+^ T-cells in CHC patients were largely indistinguishable from those of HD T-cells, the expression pattern of apoptosis-related genes in T-cells from CHC patients was dramatically altered and was sufficient to distinguish CHC patients from HDs. Consistent with the normal lymphocyte compartment but enhanced activation-induced apoptosis observed in the CHC patients, these results suggest that CHC-induced host inflammation primarily affects apoptosis. Furthermore, the expression of multiple apoptosis-related genes was dysregulated in CD4^+^ T-cells from CHC patients. The expression of some of these genes, such as BCL2A1, was also perturbed in global CD8^+^ T-cells from CHC patients. Interestingly, one-third of these genes were significantly correlated with clinical parameters in CHC patients. For example, the expression of DAPK1 was significantly positively correlated with ALT and AST values. DAPK1 is induced after T-cell stimulation and inhibits T-cells activation [42]. The expression of superoxide dismutase 2 (SOD2), a protein that inhibits radiation-induced apoptosis [43], was strongly correlated with ALT, TBIL and DBIL values, suggesting that apoptosis-related genes may contribute to pathology during HCV infection. 

Importantly, the gene signature in T-cells from CHC patients differed from those in T-cells from HIV-infected and HBV-infected patients. The gene signature of CD4^+^ T-cells from HIV-infected patients is characterized by the induction of ISGs [10,11]. We recently demonstrated that three apoptosis-related genes –Apoptotic protease-activating factor 1 (APAF1), mitogen-activated protein kinase 4 (MAP2K4), and tumor protein p53 (TP53) – were upregulated in PBMCs from HIV rapid progressors [44]. Similarly, CD8^+^ T-cells from HIV-1 infected patients also exhibit upregulation of ISGs. Although apoptosis is enhanced in T-cells from HIV-1 patients, the changes in the expression of apoptosis-related genes are different from those observed in CHC patients, suggesting that these changes are virus-specific. Thus, specific strategies are needed to prevent virus-induced T-cells loss in chronic HCV infection.

In conclusion, our data suggest that activation-induced apoptosis is a primary functional failure for global CD4^+^ and CD8^+^ T-cells in long-term chronic HCV infection, accompanying with unique apoptosis-related gene expression signature. Several cytokines elevated in the HCV patients’ plasma, including IFN-γ, IP-10 and IL-9, are responsible for sensitization of global T-cells to activation-induced cell death. However, it remains to be investigated whether these cytokines directly lead antigen-specific T-cells to activation-induced apoptosis. 

## Supporting Information

Figure S1 **Correlations between the HCV RNA titer and other clinical parameters among the CHC patients.** Shown are the correlations between HCV RNA titers and ALT, AST, DBIL, TBIL, age and gender. None has achieved statistic significance.
(TIF)

Figure S2 **Clustering analysis of genes with CV>0.5.** (A), Clustering result for CD4^+^ T-cells is presented. (B), Clustering result for CD8^+^ T-cells is presented. The blocks in blue represent HD samples, the blocks in yellow represent HCV-l samples, and the block in red represents HCV-h samples.
(TIF)

Figure S3 **List of altered gene pathways in CD4^+^ and CD8^+^ T lymphocytes of CHC patients.** Shown are the results of pathway analysis by GeneGo for significantly changed expression of genes (fold change ≥1.5, p<0.05) of CD4^+^ (A) and CD8^+^ (B) T-cells. Note that the pathways in HCV-l and HCV-h patients differed from each other.
(TIF)

Table S1 **Genes involved in critical biological processes with up- or down-regulated expression from CD4^+^ T-cells in HCV-h or HCV-l groups compared to healthy donors.**
(DOCX)

Table S2 **Genes involved in critical biological processes with up- or down-regulated expression from CD8^+^ T-cells in HCV-h or HCV-l groups compared to healthy donors.**
(DOCX)

Table S3 **Common genes of CD4^+^ T-cells shared by HCV and HIV-1infection.**
(DOCX)

Table S4 **Common genes of CD8^+^ T-cells shared by HCV and HBV infection.**
(DOCX)

Table S5 **Common genes of CD8^+^ T-cells shared by HCV and HIV-1 infection.**
(DOCX)
