# Supplementary figures and images for: T Lymphocytes from Chronic HCV-Infected Patients Are Primed for Activation-Induced Apoptosis and Express Unique Pro-Apoptotic Gene Signature
Source: PLoS One. 2013 Oct 10;8(10):e77008. doi: 10.1371/journal.pone.0077008 (PMC3794995; doi:10.1371/journal.pone.0077008)

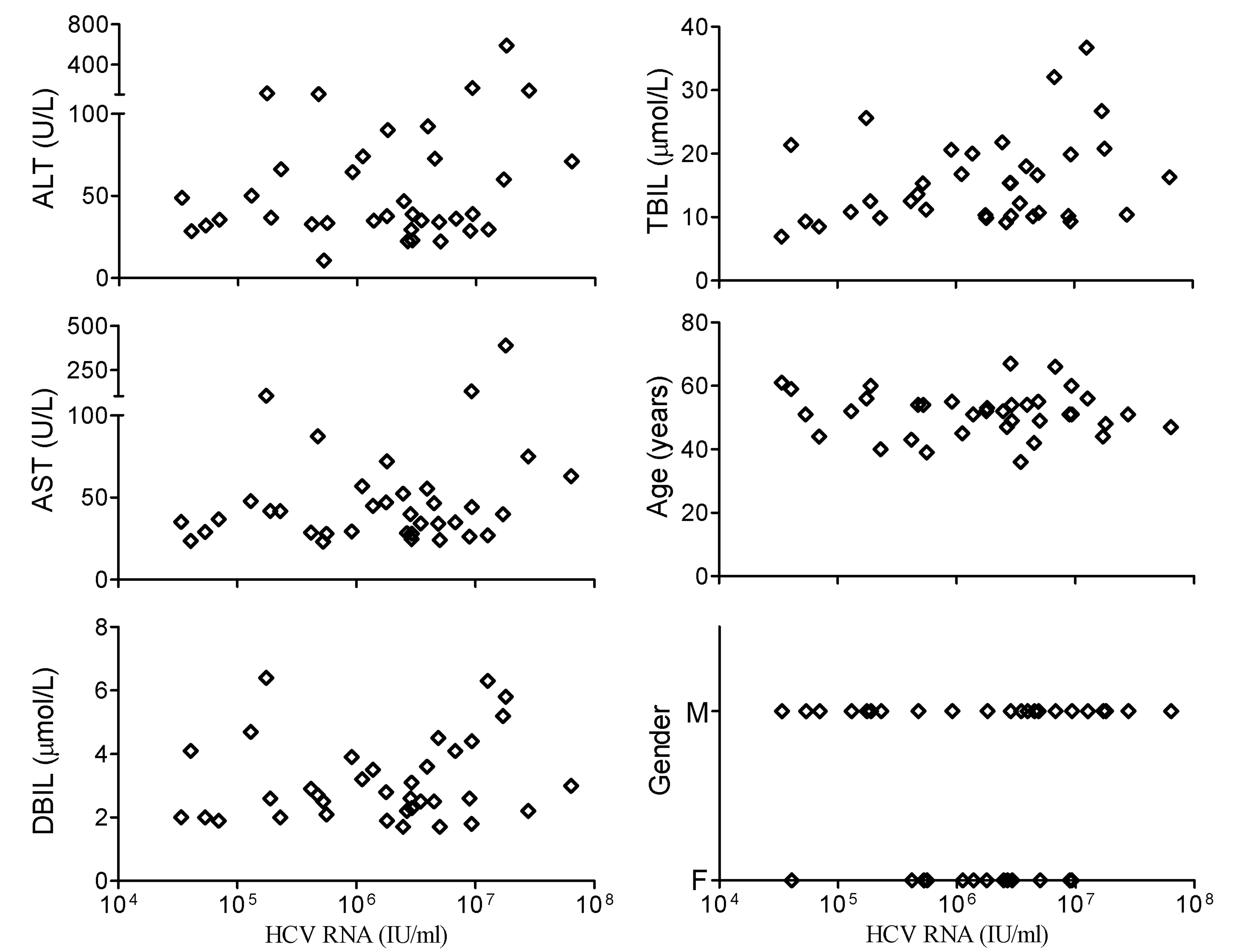

Supplement: Figure S1 — Correlations between the HCV RNA titer and other clinical parameters among the CHC patients. Shown are the correlations between HCV RNA titers and ALT, AST, DBIL, TBIL, age and gender. None has achieved statistic significance. (TIF) [file pone.0077008.s001.tif]

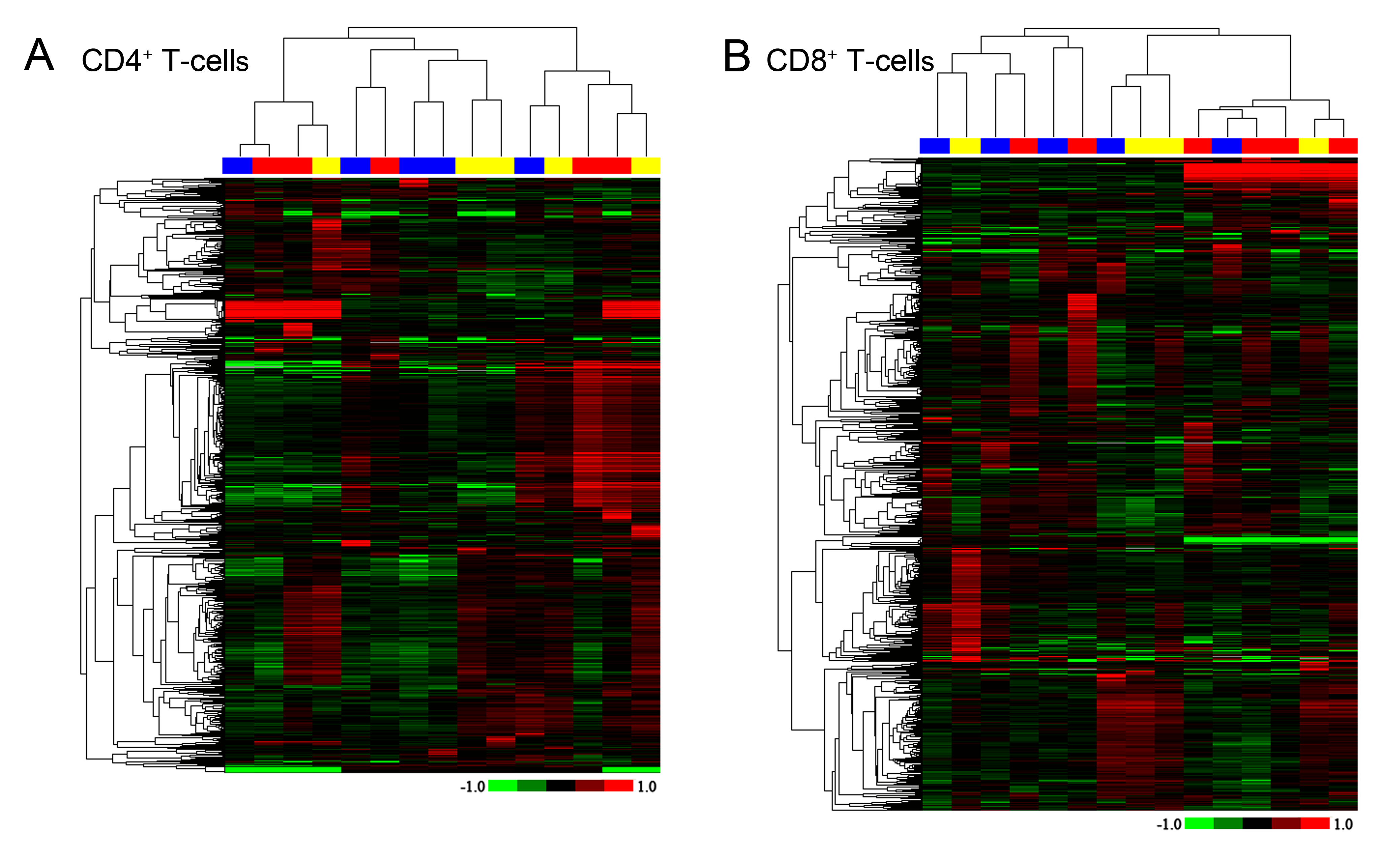

Supplement: Figure S2 — Clustering analysis of genes with CV>0.5. (A), Clustering result for CD4+ T-cells is presented. (B), Clustering result for CD8+ T-cells is presented. The blocks in blue represent HD samples, the blocks in yellow represent HCV-l samples, and the block in red represents HCV-h samples. (TIF) [file pone.0077008.s002.tif]

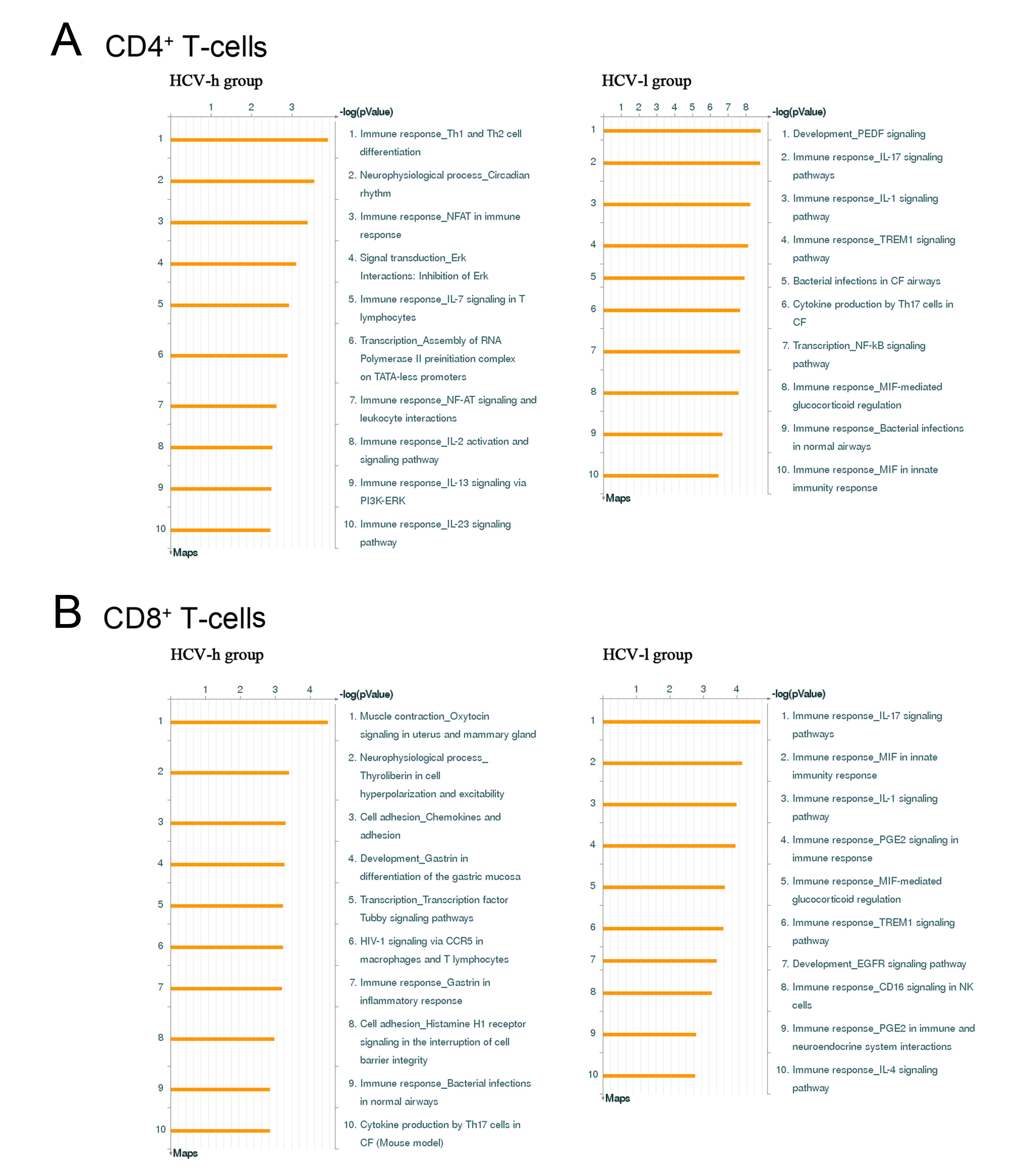

Supplement: Figure S3 — List of altered gene pathways in CD4+ and CD8+ T lymphocytes of CHC patients. Shown are the results of pathway analysis by GeneGo for significantly changed expression of genes (fold change ≥1.5, p<0.05) of CD4+ (A) and CD8+ (B) T-cells. Note that the pathways in HCV-l and HCV-h patients differed from each other. (TIF) [file pone.0077008.s003.tif]
