# Supplementary material for: T Lymphocytes from Chronic HCV-Infected Patients Are Primed for Activation-Induced Apoptosis and Express Unique Pro-Apoptotic Gene Signature
Source: PLoS One. 2013 Oct 10;8(10):e77008. doi: 10.1371/journal.pone.0077008 (PMC3794995; doi:10.1371/journal.pone.0077008)
Supplement: Table S1 — Genes involved in critical biological processes with up- or down-regulated expression from CD4+ T-cells in HCV-h or HCV-l groups compared to healthy donors. (DOCX) [file pone.0077008.s004.docx]

**Table S1. Genes involved in critical biological processes with up- or down-regulated expression from CD4^+^ T-cells in HCV-h or HCV-l groups compared to healthy donors.**

| **HCV-h group vs. HD group** | | **HCV-l group vs. HD group** | |
| --- | --- | --- | --- |
| Up-regulated | Down-regulated | Up-regulated | Down-regulated |
| ***Apoptosis*** |  |  |  |
| CASP1, RIPK2 | TNFRSF25, WWOX, STK4, STK17B, PERP, PLEKHF1 | SERPINB2, PTGS2, IRAK2, NLRP3, DRAM1, SOD2, BCL2A1, DAPK1, TNFAIP8, CASP1, PHLDA1, SERPINB9, THAP2, CFLAR, PHLDA2, CD38, PMAIP1, RNF144B, PLEKHF2, SGMS1, CLN8, RIPK2, MST4, IRC3, IRAK3, CARD16///CASP1 | WWOX, TIAM1, ZMAT3, TCTN3, BRE, CYFIP2, PPP3R1, FEM1B, CARD14, FOXO1, BCL2, ITPR1 |
| ***Cytokines, chemokines and receptors*** | |  |  |
| CXCL10, IL13RA1, CSF2RB, CXCL9 | IL18R1, CCR6 | CXCL3, IL6, IL1A, CCL4, CCL20, IL1RN, CXCL2, CCL3 /// CCL3L1 /// CCL3L3, TNF, IL1R1, IL8, CSF2, IFNG, IL1B, IL1RAP, IFNGR2, IL23A, CCL7, IL3RA, CXCL1, CSF3 | CCR6 |
| ***Signal transduction, kinase cascade*** | |  |  |
| ZAK, ARHGEF10, LRRN3 | STAT4, JAK1, NFATC2, SOCS2, LTK, PLEKHA1,MAP3K4, TNIK, DUSP16, MCTP2, RPS6KA3, DUSP1, CHN1, RASGEF1A, MYO6 | GPR84, RAPGEF2, RIN2, SDC4, FFAR2, ZAK, ACVR2A, TBC1D12, ADORA2A /// CYTSA, SPRED2, NFKBID, NFKB1, ADRB2, DUSP6, ARL5B, MAPK6, MAP3K8, MAPK8, ARL8B, NFKBIZ, RAP2C, APLP2, HMGCR, TANK, RASGEF1B, RAB38, SPAG9, MAP2K3, NRAS | ACVR2B, PPM1L, MAP4K1, ERC1, SRPK2, GIT2, NCOA2, PRKCQ, TRIB2, MAP3K3, TAS2R14, FOS, SOCS2, TBC1D1, CORO2A, MAP2K5, MAP3K4, ACAP2, CBL, MAPK1, DUSP7, MCTP2 |
| ***Costimulatory and inhibitory receptors*** | |  |  |
|  | TNFRSF4 | CD40, TNFRSF9 | CD27 |
