# Supplementary material for: T Lymphocytes from Chronic HCV-Infected Patients Are Primed for Activation-Induced Apoptosis and Express Unique Pro-Apoptotic Gene Signature
Source: PLoS One. 2013 Oct 10;8(10):e77008. doi: 10.1371/journal.pone.0077008 (PMC3794995; doi:10.1371/journal.pone.0077008)
Supplement: Table S2 — Genes involved in critical biological processes with up- or down-regulated expression from CD8+ T-cells in HCV-h or HCV-l groups compared to healthy donors. (DOCX) [file pone.0077008.s005.docx]

**Table S2. Genes involved in critical biological processes with up- or down-regulated expression from CD8^+^ T-cells in HCV-h or HCV-l groups compared to healthy donors.**

| **HCV-h group vs. HD group** | | **HCV-l group vs. HD group** | |
| --- | --- | --- | --- |
| Up-regulated | Down-regulated | Up-regulated | Down-regulated |
| ***Apoptosis*** |  |  |  |
| PTGS2, MAL, IGF1R, HIPK2, BCL2A1, HIPK1 | PERP, ZBTB16, | TGS2, SERPINB2, NLRP3, IER3, ARHGEF7, BCLAF1, SOD2, BCL2A1 | GSK3B, TNFRSF1A, ZBTB16 |
| ***Cytokines, chemokines and receptors*** | |  |  |
| IL8, IL1RN, IL6R, CXCL2, CSF2RB, CSF3R, IL1R2 | CCR5, IL12RB2, IL18R1, IL18RAP, CXCR6 | IL8, IL1B, CXCL2, IL1RN, IFNG, CCL4, IL1A | IL23R |
| ***Signal transduction, kinase cascade*** | |  |  |
| LRRN3, ARHGEF10, PRKCA, NFKBID, RCAN3, ACVR2A, FFAR2, PLEKHG4 | PRKCB, LATS2, PTPN22, SMAD5, ADRB2, SIPA1L2, MCTP2, S1PR5, AGAP1, CD101, MAP3K4, LTK, KLRB1 | RHEB, MFSD6, ASAP1, MAPK8, RABGAP1L | SMAD5, CBL, MCTP2, SIPA1L2, CD101, WDR67, DUSP1, TAS2R14 |
| ***Costimulatory and inhibitory receptors*** | |  |  |
|  | KIR3DL1 /// KIR3DL2 /// LOC727787 |  |  |
