# Supplementary material for: T Lymphocytes from Chronic HCV-Infected Patients Are Primed for Activation-Induced Apoptosis and Express Unique Pro-Apoptotic Gene Signature
Source: PLoS One. 2013 Oct 10;8(10):e77008. doi: 10.1371/journal.pone.0077008 (PMC3794995; doi:10.1371/journal.pone.0077008)
Supplement: Table S3 — Common genes of CD4+ T-cells shared by HCV and HIV-1infection. (DOCX) [file pone.0077008.s006.docx]

| **Table S3. Common genes of CD4^+^ T-cells shared by HCV and HIV-1 infection.** | | | |  |  |  |  |  |
| --- | --- | --- | --- | --- | --- | --- | --- | --- |
| **Gene Symbol** | **Probeset ID** | **Gene Title** | **Transcript ID** | | **p-value** | **Ratio** | **Ratio (Description)** | **Reference** |
| ADORA2A | ILMN_1807372 | adenosine A2a receptor /// cytospin A | ILMN_8227 | | 2.29E-05 | 0.42 | HIV down vs HD | Ref 2 |
| ADORA2A /// CYTSA | 205013_s_at |  | NM_000675 /// NM_001145468 /// NM_015330 | | 0.0125 | 2.09 | HCV low up vs HD |  |
| ARHGAP8 | ILMN_2342851 | Rho GTPase activating protein 8 /// proline rich 5 (renal) /// PRR5-ARHGAP8 read | ILMN_10507 | | 0.0018 | 2.49 | HIV up vs HD | Ref 2 |
| ARHGAP8 | ILMN_2412475 |  | ILMN_29217 | | 0.0001 | 2.28 | HIV up vs HD | Ref 2 |
| ARHGAP8 /// PRR5 /// PRR5-ARHGAP8 | 205980_s_at |  | NM_001017526 /// NM_001017528 /// NM_001017529 /// NM_001017530 /// NM_015366 // | | 0.0256 | 0.62 | HCV high down vs HD |  |
| ARHGAP8 /// PRR5 /// PRR5-ARHGAP8 | 37117_at |  | NM_001017526 /// NM_001017528 /// NM_001017529 /// NM_001017530 /// NM_015366 // | | 0.0273 | 0.64 | HCV high down vs HD |  |
| ASXL2 | ILMN_1698968 | additional sex combs like 2 (Drosophila) | ILMN_7971 | | 3.81E-06 | 0.42 | HIV down vs HD | Ref 2 |
| ASXL2 | 218659_at |  | NM_018263 | | 0.0308 | 0.65 | HCV low down vs HD |  |
| BCL2 | 232210_at | B-cell CLL/lymphoma 2 | --- | | 0.0451 | 0.48 | HCV low down vs HD |  |
| BCL2 | 232614_at |  | --- | | 0.0437 | 0.51 | HCV low down vs HD |  |
| BCL2 | ILMN_2246956 |  | ILMN_171007 | | 1.94E-05 | 0.41 | HIV down vs HD | Ref 2 |
| CA5B | ILMN_1672807 |  | ILMN_20617 | | 0.0002 | 0.45 | HIV down vs HD | Ref 2 |
| CA5B | 214082_at | carbonic anhydrase VB, mitochondrial | NM_007220 | | 0.0020 | 0.64 | HCV high down vs HD |  |
| CAMTA1 | ILMN_1661940 |  | ILMN_179380 | | 0.0001 | 0.40 | HIV down vs HD | Ref 2 |
| CAMTA1 | 1555370_a_at | calmodulin binding transcription activator 1 | NM_015215 | | 0.0004 | 0.42 | HCV down vs HD |  |
| CAMTA1 | 213268_at |  | NM_015215 | | 0.0007 | 0.39 | HCV down vs HD |  |
| CAMTA1 | 1555370_a_at |  | NM_015215 | | 0.0041 | 0.40 | HCV high down vs HD |  |
| CAMTA1 | 213268_at |  | NM_015215 | | 0.0037 | 0.35 | HCV high down vs HD |  |
| CAMTA1 | 1555370_a_at |  | NM_015215 | | 0.0158 | 0.44 | HCV low down vs HD |  |
| CAMTA1 | 213268_at |  | NM_015215 | | 0.0244 | 0.43 | HCV low down vs HD |  |
| CASP1 | ILMN_2326509 | caspase 1, apoptosis-related cysteine peptidase (interleukin 1, beta, convertase | ILMN_10621 | | 4.25E-05 | 0.40 | HIV down vs HD | Ref 2 |
| CASP1 | 206011_at |  | NM_001223 /// NM_033292 /// NM_033293 /// NM_033294 /// NM_033295 | | 0.0096 | 1.79 | HCV high up vs HD |  |
| CASP1 | 206011_at |  | NM_001223 /// NM_033292 /// NM_033293 /// NM_033294 /// NM_033295 | | 0.0220 | 1.83 | HCV low up vs HD |  |
| CASP1 | 206011_at |  | NM_001223 /// NM_033292 /// NM_033293 /// NM_033294 /// NM_033295 | | 0.0052 | 1.71 | HCV up vs HD |  |
| CCL3 | ILMN_1671509 | chemokine (C-C motif) ligand 3 /// chemokine (C-C motif) ligand 3-like 1 /// che | ILMN_1999 | | 0.0003 | 5.92 | HIV up vs HD | Ref 2 |
| CCL3 /// CCL3L1 /// CCL3L3 | 205114_s_at |  | NM_001001437 /// NM_002983 /// NM_021006 | | 0.0122 | 2.97 | HCV low up vs HD |  |
| CCL3L3 | ILMN_2105573 |  | ILMN_22857 | | 2.69E-06 | 5.85 | HIV up vs HD | Ref 2 |
| CD38 | 236191_at | CD38 molecule | --- | | 0.0264 | 1.79 | HCV low up vs HD |  |
| CD38 | ILMN_2233783 |  | ILMN_8672 | | 4.82E-05 | 0.46 | HIV down vs HD | Ref 2 |
| CD83 | ILMN_1780582 | CD83 molecule | ILMN_166338 | | 2.14E-05 | 2.16 | HIV up vs HD | Ref 2 |
| CD83 | ILMN_2328666 |  | ILMN_181889 | | 3.53E-05 | 2.16 | HIV up vs HD | Ref 2 |
| CD83 | 204440_at |  | NM_001040280 /// NM_004233 | | 0.0084 | 1.70 | HCV low up vs HD |  |
| CLCN3 | ILMN_1660837 | chloride channel 3 | ILMN_12827 | | 2.95E-06 | 0.43 | HIV down vs HD | Ref 2 |
| CLCN3 | ILMN_1707467 |  | ILMN_12827 | | 1.17E-05 | 0.48 | HIV down vs HD | Ref 2 |
| CLCN3 | 201733_at |  | NM_001829 /// NM_173872 | | 0.0054 | 0.63 | HCV low down vs HD |  |
| CREM | 241740_at | cAMP responsive element modulator | --- | | 0.0248 | 0.61 | HCV high down vs HD |  |
| CREM | 207630_s_at |  | NM_001881 /// NM_181571 /// NM_182717 /// NM_182718 /// NM_182719 /// NM_182720 /// NM_ | | 0.0271 | 0.44 | Acute HIV down vs HD | Ref 1 |
| DDX58 | ILMN_1797001 | DEAD (Asp-Glu-Ala-Asp) box polypeptide 58 | ILMN_26258 | | 4.12E-05 | 0.44 | HIV down vs HD | Ref 2 |
| DDX58 | 242961_x_at |  | NM_014314 | | 0.0089 | 2.02 | HCV high up vs HD |  |
| DDX58 | 242961_x_at |  | NM_014314 | | 0.0075 | 1.78 | HCV up vs HD |  |
| EPB41 | 236379_at | erythrocyte membrane protein band 4.1 (elliptocytosis 1, RH-linked) | --- | | 0.0024 | 0.61 | HCV low down vs HD |  |
| EPB41 | ILMN_1663786 |  | ILMN_165423 | | 0.0001 | 0.49 | HIV down vs HD | Ref 2 |
| FCAR | ILMN_2365091 | Fc fragment of IgA, receptor for | ILMN_8722 | | 0.0006 | 3.51 | HIV up vs HD | Ref 2 |
| FCAR | 207674_at |  | NM_002000 /// NM_133269 /// NM_133271 /// NM_133272 /// NM_133273 /// NM_133274 | | 0.0442 | 1.56 | HCV high up vs HD |  |
| FCAR | 211306_s_at |  | NM_002000 /// NM_133269 /// NM_133271 /// NM_133272 /// NM_133273 /// NM_133274 | | 0.0422 | 1.61 | HCV high up vs HD |  |
| FCAR | 207674_at |  | NM_002000 /// NM_133269 /// NM_133271 /// NM_133272 /// NM_133273 /// NM_133274 | | 0.0355 | 1.54 | HCV up vs HD |  |
| FLJ90757 | ILMN_1758642 | hypothetical LOC440465 | ILMN_175182 | | 0.0001 | 0.35 | HIV down vs HD | Ref 2 |
| FLJ90757 | 1566558_x_at |  | NR_026857 | | 0.0172 | 0.66 | HCV down vs HD |  |
| FLJ90757 | 1566558_x_at |  | NR_026857 | | 0.0005 | 0.53 | HCV low down vs HD |  |
| FLJ90757 | 1566557_at |  | NR_026857 | | 0.0037 | 0.48 | HCV low down vs HD |  |
| G0S2 | ILMN_1691846 | G0/G1switch 2 | ILMN_21428 | | 0.0122 | 2.33 | HIV up vs HD | Ref 2 |
| G0S2 | 213524_s_at |  | NM_015714 | | 0.0234 | 1.80 | HCV low up vs HD |  |
| GALK2 | 230918_at | galactokinase 2 | --- | | 0.0449 | 0.58 | HCV low down vs HD |  |
| GALK2 | ILMN_1723124 |  | ILMN_181436 | | 0.0002 | 0.48 | HIV down vs HD | Ref 2 |
| GBP1 | 202269_x_at | guanylate binding protein 1, interferon-inducible, 67kDa | NM_002053 | | 0.0094 | 2.83 | Acute HIV up vs HD | Ref 1 |
| GBP1 | 202270_at |  | NM_002053 | | 0.0021 | 4.12 | Acute HIV up vs HD | Ref 1 |
| GBP1 | 202270_at |  | NM_002053 | | 0.0306 | 2.69 | Chronic HIV up vs HD | Ref 1 |
| GBP1 | 202269_x_at |  | NM_002053 | | 0.0148 | 3.02 | Chronic HIV up vs HD | Ref 1 |
| GBP1 | 202270_at |  | NM_002053 | | 0.0403 | 2.22 | HCV high up vs HD |  |
| GBP1 | 231578_at |  | NM_002053 | | 0.0025 | 1.65 | HCV low up vs HD |  |
| GBP1 | 202270_at |  | NM_002053 | | 0.0147 | 2.72 | HCV low up vs HD |  |
| GBP1 | 202270_at |  | NM_002053 | | 0.0136 | 2.23 | HCV up vs HD |  |
| GBP1 | 231577_s_at |  | NM_002053 | | 0.0368 | 1.82 | HCV up vs HD |  |
| GEM | ILMN_2367883 | GTP binding protein overexpressed in skeletal muscle | ILMN_16170 | | 0.0007 | 4.67 | HIV up vs HD | Ref 2 |
| GEM | 204472_at |  | NM_005261 /// NM_181702 | | 0.0152 | 1.67 | HCV low up vs HD |  |
| GM2A | ILMN_2221046 | GM2 ganglioside activator | ILMN_8836 | | 1.52E-05 | 0.40 | HIV down vs HD | Ref 2 |
| GM2A | 212737_at |  | NM_000405 /// NM_001167607 | | 0.0209 | 1.57 | HCV high up vs HD |  |
| GM2A | 235678_at |  | NM_000405 /// NM_001167607 | | 0.0164 | 1.61 | HCV high up vs HD |  |
| GM2A | 35820_at |  | NM_000405 /// NM_001167607 | | 0.0164 | 1.59 | HCV high up vs HD |  |
| HDHD1A | 232974_at | haloacid dehalogenase-like hydrolase domain containing 1A | --- | | 0.0160 | 0.58 | HCV low down vs HD |  |
| HDHD1A | ILMN_1710136 |  | ILMN_165827 | | 4.43E-06 | 0.50 | HIV down vs HD | Ref 2 |
| IL1B | ILMN_1775501 | interleukin 1, beta | ILMN_27277 | | 0.0050 | 5.82 | HIV up vs HD | Ref 2 |
| IL1B | 39402_at |  | NM_000576 | | 0.0027 | 2.19 | HCV low up vs HD |  |
| IL1B | 205067_at |  | NM_000576 | | 0.0036 | 2.11 | HCV low up vs HD |  |
| IL8 | ILMN_2184373 | interleukin 8 | ILMN_179575 | | 0.0002 | 6.64 | HIV up vs HD | Ref 2 |
| IL8 | 211506_s_at |  | NM_000584 | | 0.0075 | 2.72 | HCV low up vs HD |  |
| IL8 | 202859_x_at |  | NM_000584 | | 0.0092 | 1.65 | HCV low up vs HD |  |
| LARS | ILMN_1757317 | leucyl-tRNA synthetase | ILMN_174075 | | 4.18E-06 | 0.38 | HIV down vs HD | Ref 2 |
| LARS | 223888_s_at |  | NM_020117 | | 0.0220 | 0.64 | HCV low down vs HD |  |
| LOC401233 | ILMN_1674285 | similar to HIV TAT specific factor 1; cofactor required for Tat activation of HI | ILMN_182711 | | 4.36E-05 | 0.26 | HIV down vs HD | Ref 2 |
| LOC401233 | 1558882_at |  | XR_040702 /// XR_040703 /// XR_040704 | | 0.0191 | 1.56 | HCV low up vs HD |  |
| LRFN3 | ILMN_2103919 | leucine rich repeat and fibronectin type III domain containing 3 | ILMN_16840 | | 0.0003 | 0.42 | HIV down vs HD | Ref 2 |
| LRFN3 | 219346_at |  | NM_024509 | | 0.0204 | 0.64 | HCV down vs HD |  |
| LRFN3 | 219346_at |  | NM_024509 | | 0.0121 | 0.59 | HCV low down vs HD |  |
| LRRN3 | ILMN_2048591 | leucine rich repeat neuronal 3 | ILMN_174401 | | 2.11E-05 | 0.33 | HIV down vs HD | Ref 2 |
| LRRN3 | 209841_s_at |  | NM_001099658 /// NM_001099660 /// NM_018334 | | 0.0369 | 1.55 | HCV high up vs HD |  |
| LRRN3 | 209841_s_at |  | NM_001099658 /// NM_001099660 /// NM_018334 | | 0.0131 | 1.56 | HCV up vs HD |  |
| MAP3K8 | ILMN_1741159 | mitogen-activated protein kinase kinase kinase 8 | ILMN_17248 | | 1.02E-05 | 2.20 | HIV up vs HD | Ref 2 |
| MAP3K8 | 205027_s_at |  | NM_005204 | | 0.0313 | 1.56 | HCV high up vs HD |  |
| MAP3K8 | 205027_s_at |  | NM_005204 | | 0.0099 | 1.72 | HCV low up vs HD |  |
| MAP3K8 | 205027_s_at |  | NM_005204 | | 0.0067 | 1.59 | HCV up vs HD |  |
| MARCH9 | ILMN_1748393 | membrane-associated ring finger (C3HC4) 9 | ILMN_16648 | | 4.24E-05 | 0.50 | HIV down vs HD | Ref 2 |
| MARCH9 | 226454_at |  | NM_138396 | | 0.0020 | 0.64 | HCV down vs HD |  |
| MARCH9 | 226454_at |  | NM_138396 | | 0.0009 | 0.61 | HCV low down vs HD |  |
| MGC40069 | 243602_at | Hypothetical protein MGC40069 | --- | | 0.0049 | 0.65 | HCV high down vs HD |  |
| MGC40069 | ILMN_1664248 |  | ILMN_30193 | | 0.0000 | 0.34 | HIV down vs HD | Ref 2 |
| MIB1 | ILMN_1788832 | mindbomb homolog 1 (Drosophila) | ILMN_5164 | | 0.0001 | 0.42 | HIV down vs HD | Ref 2 |
| MIB1 | 224722_at |  | NM_020774 | | 0.0295 | 0.64 | HCV low down vs HD |  |
| MSC | ILMN_1741404 | musculin | ILMN_14655 | | 0.0025 | 2.33 | HIV up vs HD | Ref 2 |
| MSC | 209928_s_at |  | NM_005098 | | 0.0284 | 1.77 | HCV low up vs HD |  |
| MTSS1 | 241154_x_at | metastasis suppressor 1 | --- | | 0.0021 | 2.44 | HCV low up vs HD |  |
| MTSS1 | 232757_at |  | --- | | 0.0285 | 1.85 | HCV low up vs HD |  |
| MTSS1 | ILMN_2073289 |  | ILMN_166688 | | 0.0002 | 0.46 | HIV down vs HD | Ref 2 |
| NR4A3 | ILMN_1807298 | nuclear receptor subfamily 4, group A, member 3 | ILMN_26721 | | 1.27E-05 | 2.63 | HIV up vs HD | Ref 2 |
| NR4A3 | ILMN_1781812 |  | ILMN_804 | | 0.0021 | 2.01 | HIV up vs HD | Ref 2 |
| NR4A3 | 216979_at |  | NM_006981 /// NM_173198 /// NM_173199 /// NM_173200 | | 0.0146 | 0.63 | HCV low down vs HD |  |
| PDE4DIP | ILMN_1749639 | phosphodiesterase 4D interacting protein | ILMN_164366 | | 0.0002 | 0.47 | HIV down vs HD | Ref 2 |
| PDE4DIP | 212390_at |  | NM_001002810 /// NM_001002811 /// NM_001002812 /// NM_014644 /// NM_022359 | | 0.0064 | 0.57 | HCV down vs HD |  |
| PDE4DIP | 214130_s_at |  | NM_001002810 /// NM_001002811 /// NM_001002812 /// NM_014644 /// NM_022359 | | 0.0267 | 0.66 | HCV down vs HD |  |
| PDE4DIP | 212390_at |  | NM_001002810 /// NM_001002811 /// NM_001002812 /// NM_014644 /// NM_022359 | | 0.0432 | 0.58 | HCV high down vs HD |  |
| PDE4DIP | 214130_s_at |  | NM_001002810 /// NM_001002811 /// NM_001002812 /// NM_014644 /// NM_022359 | | 0.0103 | 0.58 | HCV high down vs HD |  |
| PDE4DIP | 212390_at |  | NM_001002810 /// NM_001002811 /// NM_001002812 /// NM_014644 /// NM_022359 | | 0.0053 | 0.56 | HCV low down vs HD |  |
| PDE4DIP | 211751_at |  | NM_001002810 /// NM_001002811 /// NM_001002812 /// NM_014644 /// NM_022359 | | 0.0006 | 3.26 | HCV low up vs HD |  |
| PDE4DIP | 215575_at |  | NM_001002810 /// NM_001002811 /// NM_001002812 /// NM_014644 /// NM_022359 | | 0.0046 | 1.85 | HCV low up vs HD |  |
| PDE4DIP | 205872_x_at |  | NM_001002810 /// NM_001002811 /// NM_001002812 /// NM_014644 /// NM_022359 | | 0.0198 | 3.01 | HCV low up vs HD |  |
| PDE4DIP | 210305_at |  | NM_001002810 /// NM_001002811 /// NM_001002812 /// NM_014644 /// NM_022359 | | 0.0426 | 1.65 | HCV low up vs HD |  |
| PHLDA1 | 217999_s_at | pleckstrin homology-like domain, family A, member 1 | NM_007350 | | 0.0290 | 2.01 | HCV low up vs HD |  |
| PHLDA1 | 217996_at |  | NM_007350 | | 0.0157 | 1.78 | HCV low up vs HD |  |
| PHLDA1 | 217997_at |  | NM_007350 | | 0.0165 | 1.91 | HCV low up vs HD |  |
| PHLDA1 | 218000_s_at |  | NM_007350 | | 0.0169 | 1.51 | HCV low up vs HD |  |
| PKP4 | ILMN_1749410 | plakophilin 4 | ILMN_11784 | | 1.54E-05 | 0.50 | HIV down vs HD | Ref 2 |
| PKP4 | 201928_at |  | NM_001005476 /// NM_003628 | | 0.0087 | 0.64 | HCV low down vs HD |  |
| PKP4 | 201929_s_at |  | NM_001005476 /// NM_003628 | | 0.0105 | 0.65 | HCV low down vs HD |  |
| PMAIP1 | ILMN_2098446 | phorbol-12-myristate-13-acetate-induced protein 1 | ILMN_25637 | | 0.0001 | 2.63 | HIV up vs HD | Ref 2 |
| PMAIP1 | 204286_s_at |  | NM_021127 | | 0.0206 | 1.76 | HCV low up vs HD |  |
| PPP1R16B | 243771_at | protein phosphatase 1, regulatory (inhibitor) subunit 16B | --- | | 0.0092 | 0.64 | HCV down vs HD |  |
| PPP1R16B | 1563473_at |  | --- | | 0.0105 | 0.57 | HCV down vs HD |  |
| PPP1R16B | 243771_at |  | --- | | 0.0050 | 0.57 | HCV high down vs HD |  |
| PPP1R16B | 1563473_at |  | --- | | 0.0099 | 0.56 | HCV low down vs HD |  |
| PPP1R16B | ILMN_1727098 |  | ILMN_26824 | | 1.75E-05 | 2.57 | HIV up vs HD | Ref 2 |
| PPP1R16B | 233813_at |  | NM_001172735 /// NM_015568 | | 0.0269 | 0.65 | HCV down vs HD |  |
| PPP1R16B | 212750_at |  | NM_001172735 /// NM_015568 | | 0.0188 | 0.62 | HCV high down vs HD |  |
| PPP1R16B | 233813_at |  | NM_001172735 /// NM_015568 | | 0.0480 | 0.63 | HCV high down vs HD |  |
| PPP1R16B | 41577_at |  | NM_001172735 /// NM_015568 | | 0.0236 | 0.59 | HCV high down vs HD |  |
| PRG2 | ILMN_1729314 | proteoglycan 2, bone marrow (natural killer cell activator, eosinophil granule m | ILMN_25091 | | 1.64E-05 | 3.00 | HIV up vs HD | Ref 2 |
| PRG2 | 211743_s_at |  | NM_002728 | | 0.0342 | 2.01 | HCV low up vs HD |  |
| PRKX | 204061_at | protein kinase, X-linked | NM_005044 | | 0.0299 | 0.64 | HCV down vs HD |  |
| PRKX | 204061_at |  | NM_005044 | | 0.0061 | 0.56 | HCV high down vs HD |  |
| PRKY | ILMN_1772163 |  | ILMN_32106 | | 2.00E-06 | 3.19 | HIV up vs HD | Ref 2 |
| PTGS2 | ILMN_2054297 | prostaglandin-endoperoxide synthase 2 (prostaglandin G/H synthase and cyclooxyge | ILMN_176524 | | 1.38E-05 | 2.63 | HIV up vs HD | Ref 2 |
| PTGS2 | 204748_at |  | NM_000963 | | 0.0017 | 4.47 | HCV low up vs HD |  |
| PTGS2 | 1554997_a_at |  | NM_000963 | | 0.0018 | 5.68 | HCV low up vs HD |  |
| RAB22A | 241301_at | RAB22A, member RAS oncogene family | --- | | 0.0114 | 0.66 | HCV low down vs HD |  |
| RAB22A | ILMN_1786976 |  | ILMN_27304 | | 3.73E-05 | 0.48 | HIV down vs HD | Ref 2 |
| RNASE6 | ILMN_1780533 | ribonuclease, RNase A family, k6 | ILMN_14848 | | 0.0001 | 0.41 | HIV down vs HD | Ref 2 |
| RNASE6 | 213566_at |  | NM_005615 | | 0.0094 | 1.74 | HCV high up vs HD |  |
| RNF144B | ILMN_1752526 | Ring finger protein 144B | ILMN_9298 | | 0.0001 | 0.36 | HIV down vs HD | Ref 2 |
| RNF144B | 239012_at |  | NM_182757 | | 0.0304 | 1.72 | HCV low up vs HD |  |
| SAMSN1 | ILMN_2171289 | SAM domain, SH3 domain and nuclear localization signals 1 | ILMN_7778 | | 0.0003 | 2.06 | HIV up vs HD | Ref 2 |
| SAMSN1 | 1555638_a_at |  | NM_022136 | | 0.0043 | 1.80 | HCV low up vs HD |  |
| SAMSN1 | 1569599_at |  | NM_022136 | | 0.0025 | 1.64 | HCV low up vs HD |  |
| SAMSN1 | 220330_s_at |  | NM_022136 | | 0.0077 | 1.51 | HCV low up vs HD |  |
| SASH1 | ILMN_2185984 | SAM and SH3 domain containing 1 | ILMN_181709 | | 0.0002 | 0.26 | HIV down vs HD | Ref 2 |
| SASH1 | 213236_at |  | NM_015278 | | 0.0293 | 1.50 | HCV high up vs HD |  |
| SCML4 | ILMN_1747436 | sex comb on midleg-like 4 (Drosophila) | ILMN_18691 | | 4.01E-05 | 0.42 | HIV down vs HD | Ref 2 |
| SCML4 | 1569225_a_at |  | NM_198081 | | 0.0243 | 0.63 | HCV low down vs HD |  |
| SERPINB2 | ILMN_2150856 | serpin peptidase inhibitor, clade B (ovalbumin), member 2 | ILMN_14466 | | 4.42E-07 | 2.37 | HIV up vs HD | Ref 2 |
| SERPINB2 | 204614_at |  | NM_001143818 /// NM_002575 | | 0.0011 | 8.62 | HCV low up vs HD |  |
| SLC38A9 | ILMN_1773643 | solute carrier family 38, member 9 | ILMN_10221 | | 3.50E-05 | 0.50 | HIV down vs HD | Ref 2 |
| SLC38A9 | 243709_at |  | NM_173514 | | 0.0185 | 0.59 | HCV low down vs HD |  |
| SOCS2 | ILMN_2131861 | suppressor of cytokine signaling 2 | ILMN_4851 | | 1.86E-05 | 0.37 | HIV down vs HD | Ref 2 |
| SOCS2 | 203372_s_at |  | NM_003877 | | 0.0023 | 0.64 | HCV down vs HD |  |
| SOCS2 | 203372_s_at |  | NM_003877 | | 0.0302 | 0.66 | HCV high down vs HD |  |
| SOCS2 | 203372_s_at |  | NM_003877 | | 0.0079 | 0.62 | HCV low down vs HD |  |
| SRGAP2 | 232095_at | SLIT-ROBO Rho GTPase activating protein 2 | --- | | 0.0387 | 0.66 | HCV low down vs HD |  |
| SRGAP2 | ILMN_1759549 |  | ILMN_7259 | | 0.0037 | 0.47 | HIV down vs HD | Ref 2 |
| SRGAP2 | 1556203_a_at |  | NM_001042758 /// NM_001170637 /// NM_015326 | | 0.0326 | 0.52 | HCV low down vs HD |  |
| ST8SIA1 | ILMN_2048011 | ST8 alpha-N-acetyl-neuraminide alpha-2,8-sialyltransferase 1 | ILMN_181880 | | 1.19E-05 | 0.42 | HIV down vs HD | Ref 2 |
| ST8SIA1 | 210073_at |  | NM_003034 | | 0.0463 | 0.53 | HCV low down vs HD |  |
| TARP | 209813_x_at | TCR gamma alternate reading frame protein | NM_001003799 /// NM_001003806 | | 0.0180 | 2.28 | Chronic HIV up vs HD | Ref 1 |
| TARP | 209813_x_at |  | NM_001003799 /// NM_001003806 | | 0.0220 | 0.64 | HCV high down vs HD |  |
| TARP /// TRGC2 | 211144_x_at | TCR gamma alternate reading frame protein /// T cell receptor gamma constant 2 | NM_001003799 /// NM_001003806 | | 0.0235 | 2.31 | Chronic HIV up vs HD | Ref 1 |
| TARP /// TRGC2 | 215806_x_at |  | NM_001003799 /// NM_001003806 | | 0.0184 | 2.28 | Chronic HIV up vs HD | Ref 1 |
| TARP /// TRGC2 | 216920_s_at |  | NM_001003799 /// NM_001003806 | | 0.0319 | 2.17 | Chronic HIV up vs HD | Ref 1 |
| TARP /// TRGC2 | 216920_s_at |  | NM_001003799 /// NM_001003806 | | 0.0445 | 0.63 | HCV down vs HD |  |
| TARP /// TRGC2 | 211144_x_at |  | NM_001003799 /// NM_001003806 | | 0.0234 | 0.65 | HCV high down vs HD |  |
| TARP /// TRGC2 | 215806_x_at |  | NM_001003799 /// NM_001003806 | | 0.0287 | 0.65 | HCV high down vs HD |  |
| TARP /// TRGC2 | 216920_s_at |  | NM_001003799 /// NM_001003806 | | 0.0298 | 0.59 | HCV high down vs HD |  |
| TBC1D1 | ILMN_1754947 | TBC1 (tre-2/USP6, BUB2, cdc16) domain family, member 1 | ILMN_3070 | | 2.88E-05 | 0.35 | HIV down vs HD | Ref 2 |
| TBC1D1 | 1569566_at |  | NM_015173 | | 0.0151 | 0.62 | HCV low down vs HD |  |
| THBS1 | 239336_at | thrombospondin 1 | --- | | 0.0075 | 1.69 | HCV low up vs HD |  |
| THBS1 | ILMN_1686116 |  | ILMN_182705 | | 0.0002 | 3.22 | HIV up vs HD | Ref 2 |
| THBS1 | 201109_s_at |  | NM_003246 | | 0.0260 | 4.11 | HCV low up vs HD |  |
| THBS1 | 201108_s_at |  | NM_003246 | | 0.0134 | 3.02 | HCV low up vs HD |  |
| THBS1 | 201110_s_at |  | NM_003246 | | 0.0174 | 5.41 | HCV low up vs HD |  |
| THBS1 | 235086_at |  | NM_003246 | | 0.0316 | 5.41 | HCV low up vs HD |  |
| TSPAN18 | ILMN_1699980 | tetraspanin 18 | ILMN_14181 | | 0.0002 | 0.48 | HIV down vs HD | Ref 2 |
| TSPAN18 | 227307_at |  | NM_001031730 /// NM_130783 | | 0.0233 | 0.65 | HCV low down vs HD |  |
| TTC17 | ILMN_1660810 | tetratricopeptide repeat domain 17 | ILMN_22995 | | 0.0003 | 0.49 | HIV down vs HD | Ref 2 |
| TTC17 | 232323_s_at |  | NM_018259 | | 0.0163 | 0.65 | HCV down vs HD |  |
| TTC17 | 232323_s_at |  | NM_018259 | | 0.0400 | 0.65 | HCV low down vs HD |  |
| UBTD2 | ILMN_1731412 | ubiquitin domain containing 2 | ILMN_6162 | | 7.18E-06 | 0.31 | HIV down vs HD | Ref 2 |
| UBTD2 | 224834_at |  | NM_152277 | | 0.0003 | 1.74 | HCV low up vs HD |  |
| UBTD2 | 224827_at |  | NM_152277 | | 0.0067 | 2.13 | HCV low up vs HD |  |
| UBTD2 | 224827_at |  | NM_152277 | | 0.0483 | 1.64 | HCV up vs HD |  |
| ZDHHC11 | ILMN_1694514 | zinc finger, DHHC-type containing 11 | ILMN_25571 | | 0.0010 | 0.45 | HIV down vs HD | Ref 2 |
| ZDHHC11 | 1552283_s_at |  | NM_024786 | | 0.0115 | 0.36 | HCV high down vs HD |  |
| ZDHHC11 | 221646_s_at |  | NM_024786 | | 0.0093 | 0.34 | HCV high down vs HD |  |
| ZDHHC11 | 232417_x_at |  | NM_024786 | | 0.0329 | 0.65 | HCV high down vs HD |  |
| ZEB2 | 1557797_a_at | zinc finger E-box binding homeobox 2 | --- | | 0.0120 | 1.72 | HCV high up vs HD |  |
| ZEB2 | 1562194_at |  | --- | | 0.0152 | 1.73 | HCV high up vs HD |  |
| ZEB2 | 1557797_a_at |  | --- | | 0.0012 | 2.26 | HCV low up vs HD |  |
| ZEB2 | 1562194_at |  | --- | | 0.0011 | 2.08 | HCV low up vs HD |  |
| ZEB2 | 239296_at |  | --- | | 0.0067 | 2.18 | HCV low up vs HD |  |
| ZEB2 | 1557797_a_at |  | --- | | 0.0026 | 1.89 | HCV up vs HD |  |
| ZEB2 | 1562194_at |  | --- | | 0.0016 | 1.81 | HCV up vs HD |  |
| ZEB2 | 239296_at |  | --- | | 0.0246 | 1.81 | HCV up vs HD |  |
| ZEB2 | ILMN_1688698 |  | ILMN_14685 | | 3.84E-06 | 4.51 | HIV up vs HD | Ref 2 |
| ZEB2 | 203603_s_at |  | NM_001171653 /// NM_014795 /// NR_033258 | | 0.0268 | 1.90 | HCV high up vs HD |  |
| ZEB2 | 203603_s_at |  | NM_001171653 /// NM_014795 /// NR_033258 | | 0.0012 | 2.63 | HCV low up vs HD |  |
| ZEB2 | 228333_at |  | NM_001171653 /// NM_014795 /// NR_033258 | | 0.0076 | 2.56 | HCV low up vs HD |  |
| ZEB2 | 235593_at |  | NM_001171653 /// NM_014795 /// NR_033258 | | 0.0148 | 1.98 | HCV low up vs HD |  |
| ZEB2 | 203603_s_at |  | NM_001171653 /// NM_014795 /// NR_033258 | | 0.0029 | 2.15 | HCV up vs HD |  |
| ZFY | 1556677_at | zinc finger protein, Y-linked | --- | | 0.0174 | 1.73 | HCV high up vs HD |  |
| ZFY | ILMN_2090059 |  | ILMN_161877 | | 5.82E-06 | 2.79 | HIV up vs HD | Ref 2 |
| ZNF439 | ILMN_1792972 | zinc finger protein 439 | ILMN_2860 | | 0.0001 | 0.50 | HIV down vs HD | Ref 2 |
| ZNF439 | 236562_at |  | NM_152262 | | 0.0137 | 0.62 | HCV low down vs HD |  |
| ZNF514 | ILMN_2215211 | zinc finger protein 514 | ILMN_14476 | | 0.0001 | 0.35 | HIV down vs HD | Ref 2 |
| ZNF514 | 235729_at |  | NM_032788 | | 0.0326 | 0.63 | HCV low down vs HD |  |
|  |  |  |  | |  |  |  |  |
| Note: |  |  |  | |  |  |  |  |

Ref 1: Hyrcza, M.D., et al., Distinct transcriptional profiles in ex vivo CD4+ and CD8+ T cells are established early in human immunodeficiency virus type 1 infection and are characterized by a chronic interferon response as well as extensive transcriptional changes in CD8+ T cells. J Virol, 2007. 81(7): p. 3477-86.

Ref 2: Vigneault, F., et al., Transcriptional profiling of CD4 T cells identifies distinct subgroups of HIV-1 elite controllers. J Virol, 2011. 85(6): p. 3015-9.
