# Supplementary material for: T Lymphocytes from Chronic HCV-Infected Patients Are Primed for Activation-Induced Apoptosis and Express Unique Pro-Apoptotic Gene Signature
Source: PLoS One. 2013 Oct 10;8(10):e77008. doi: 10.1371/journal.pone.0077008 (PMC3794995; doi:10.1371/journal.pone.0077008)
Supplement: Table S4 — Common genes of CD8+ T-cells shared by HCV and HBV infection. (DOCX) [file pone.0077008.s007.docx]

| **Table S4. Common genes of CD8^+^ T-cells shared by HCV and HBV infection** | | | | | | |
| --- | --- | --- | --- | --- | --- | --- |
|  |  |  |  |  |  |  |
| **Gene Symbol** | **Probeset ID** | **Gene Title** | **Transcript ID** | **p-value** | **Ratio** | **Ratio(Description)** |
| CXCL2 | 209774_x_at | chemokine (C-X-C motif) ligand 2 | NM_002089 | 0.0095 | 0.49 | CHB down vs HD |
| CXCL2 | 209774_x_at |  | NM_002089 | 0.0362 | 2.03 | HCV high up vs HD |
| CXCL2 | 209774_x_at |  | NM_002089 | 0.0001 | 4.67 | HCV low up vs HD |
| CXCL2 | 209774_x_at |  | NM_002089 | 0.0020 | 2.94 | HCV up vs HD |
| DUSP1 | 201044_x_at | dual specificity phosphatase 1 | NM_004417 | 0.0114 | 0.37 | CHB down vs HD |
| DUSP1 | 201044_x_at |  | NM_004417 | 0.0173 | 0.56 | HCV low down vs HD |
| EZR | 208621_s_at | ezrin | NM_001111077 /// NM_003379 | 0.0352 | 0.62 | CHB down vs HD |
| EZR | 208621_s_at |  | NM_001111077 /// NM_003379 | 0.0033 | 1.63 | HCV low up vs HD |
| G0S2 | 213524_s_at | G0/G1switch 2 | NM_015714 | 0.0333 | 0.50 | CHB down vs HD |
| G0S2 | 213524_s_at |  | NM_015714 | 0.0184 | 4.20 | HCV high up vs HD |
| G0S2 | 213524_s_at |  | NM_015714 | 0.0024 | 3.70 | HCV low up vs HD |
| G0S2 | 213524_s_at |  | NM_015714 | 0.0027 | 3.98 | HCV up vs HD |
| ID1 | 208937_s_at | inhibitor of DNA binding 1, dominant negative helix-loop-helix protein | NM_002165 /// NM_181353 | 0.0085 | 0.16 | CHB down vs HD |
| ID1 | 208937_s_at |  | NM_002165 /// NM_181353 | 0.0106 | 0.51 | HCV high down vs HD |
| IER3 | 201631_s_at | immediate early response 3 | NM_003897 | 0.0024 | 0.48 | CHB down vs HD |
| IER3 | 201631_s_at |  | NM_003897 | 0.0195 | 2.08 | HCV low up vs HD |
| IER3 | 201631_s_at |  | NM_003897 | 0.0308 | 1.72 | HCV up vs HD |
| IL18RAP | 207072_at | interleukin 18 receptor accessory protein | NM_003853 | 0.0146 | 0.30 | CHB down vs HD |
| IL18RAP | 207072_at |  | NM_003853 | 0.0420 | 0.49 | HCV high down vs HD |
| IL8 | 202859_x_at | interleukin 8 | NM_000584 | 0.0016 | 0.10 | CHB down vs HD |
| IL8 | 202859_x_at |  | NM_000584 | 0.0052 | 4.76 | HCV high up vs HD |
| IL8 | 202859_x_at |  | NM_000584 | 1.09E-05 | 6.41 | HCV low up vs HD |
| IL8 | 202859_x_at |  | NM_000584 | 0.0001 | 5.04 | HCV up vs HD |
| IL8 | 211506_s_at |  | NM_000584 | 0.0005 | 0.25 | CHB down vs HD |
| IL8 | 211506_s_at |  | NM_000584 | 0.0093 | 5.57 | HCV high up vs HD |
| IL8 | 211506_s_at |  | NM_000584 | 3.59E-05 | 6.70 | HCV low up vs HD |
| IL8 | 211506_s_at |  | NM_000584 | 0.0003 | 6.17 | HCV up vs HD |
| ME1 | 204059_s_at | malic enzyme 1, NADP(+)-dependent, cytosolic | NM_002395 | 0.0414 | 0.34 | CHB down vs HD |
| ME1 | 204059_s_at |  | NM_002395 | 0.0052 | 0.22 | HCV high down vs HD |
| ME1 | 204059_s_at |  | NM_002395 | 0.0060 | 0.23 | HCV low down vs HD |
| PLCB1 | 213222_at | phospholipase C, beta 1 (phosphoinositide-specific) | NM_015192 /// NM_182734 | 0.0246 | 0.55 | CHB down vs HD |
| PLCB1 | 213222_at |  | NM_015192 /// NM_182734 | 0.0056 | 0.62 | HCV high down vs HD |
| PTX3 | 206157_at | pentraxin 3, long | NM_002852 | 0.0311 | 0.67 | CHB down vs HD |
| PTX3 | 206157_at |  | NM_002852 | 0.0136 | 1.63 | HCV high up vs HD |
| PTX3 | 206157_at |  | NM_002852 | 0.0042 | 1.86 | HCV low up vs HD |
| PTX3 | 206157_at |  | NM_002852 | 0.0011 | 1.79 | HCV up vs HD |
| SCRN1 | 201462_at | secernin 1 | NM_001145513 /// NM_001145514 /// NM_001145515 /// NM_014766 | 0.0090 | 0.46 | CHB down vs HD |
| SCRN1 | 201462_at |  | NM_001145513 /// NM_001145514 /// NM_001145515 /// NM_014766 | 0.0196 | 0.55 | HCV low down vs HD |
| TLE1 | 203221_at | transducin-like enhancer of split 1 (E(sp1) homolog, Drosophila) | NM_005077 | 0.0430 | 0.45 | CHB down vs HD |
| TLE1 | 203221_at |  | NM_005077 | 0.0465 | 0.62 | HCV high down vs HD |
| TLE1 | 203221_at |  | NM_005077 | 0.0106 | 0.58 | HCV low down vs HD |
| TLE1 | 203222_s_at |  | NM_005077 | 0.0288 | 0.51 | CHB down vs HD |
| TLE1 | 203222_s_at |  | NM_005077 | 0.0085 | 0.65 | HCV low down vs HD |
| ZBTB16 | 205883_at | zinc finger and BTB domain containing 16 | NM_001018011 /// NM_006006 | 0.0006 | 0.29 | CHB down vs HD |
| ZBTB16 | 205883_at |  | NM_001018011 /// NM_006006 | 0.0103 | 0.31 | HCV low down vs HD |
| ZNF573 | 217627_at | zinc finger protein 573 | NM_001172689 /// NM_001172690 /// NM_001172691 /// NM_001172692 /// NM_152360 | 0.0388 | 2.33 | CHB up vs HD |
| ZNF573 | 217627_at |  | NM_001172689 /// NM_001172690 /// NM_001172691 /// NM_001172692 /// NM_152360 | 0.0487 | 1.75 | HCV high up vs HD |
