# Supplementary material for: T Lymphocytes from Chronic HCV-Infected Patients Are Primed for Activation-Induced Apoptosis and Express Unique Pro-Apoptotic Gene Signature
Source: PLoS One. 2013 Oct 10;8(10):e77008. doi: 10.1371/journal.pone.0077008 (PMC3794995; doi:10.1371/journal.pone.0077008)
Supplement: Table S5 — Common genes of CD8+ T-cells shared by HCV and HIV-1 infection. (DOCX) [file pone.0077008.s008.docx]

| **Table S5. Common genes of CD8^+^ T-cells shared by HCV and HIV-1 infection** | | | | | | |  |
| --- | --- | --- | --- | --- | --- | --- | --- |
|  |  |  |  |  |  |  |  |
| **Gene Symbol** | **Probeset ID** | **Gene Title** | **RefSeq Transcript ID** | **p-value** | **Ratio** | **Ratio(Description)** | |
| ACTN1 | 208636_at | actinin, alpha 1 | NM_001102 /// NM_001130004 /// NM_001130005 | 0.0156 | 1.96 | HCV high up vs HD | |
| ACTN1 | 208636_at |  | NM_001102 /// NM_001130004 /// NM_001130005 | 0.0058 | 0.25 | acute down vs HD | |
| ACTN1 | 208636_at |  | NM_001102 /// NM_001130004 /// NM_001130005 | 0.0144 | 0.31 | choronic down vs HD | |
| ACTN1 | 208636_at |  | NM_001102 /// NM_001130004 /// NM_001130005 | 0.0263 | 0.35 | acute down vs controller | |
| ACTN1 | 208637_x_at |  | NM_001102 /// NM_001130004 /// NM_001130005 | 0.0209 | 1.91 | HCV high up vs HD | |
| ACTN1 | 208637_x_at |  | NM_001102 /// NM_001130004 /// NM_001130005 | 0.0147 | 0.48 | acute down vs HD | |
| ACTN1 | 208637_x_at |  | NM_001102 /// NM_001130004 /// NM_001130005 | 0.0034 | 0.40 | choronic down vs HD | |
| CCR5 | 206991_s_at | chemokine (C-C motif) receptor 5 | NM_000579 /// NM_001100168 | 0.0424 | 0.59 | HCV high down vs HD | |
| CCR5 | 206991_s_at |  | NM_000579 /// NM_001100168 | 8.49E-07 | 4.34 | acute up vs HD | |
| CCR5 | 206991_s_at |  | NM_000579 /// NM_001100168 | 0.0002 | 2.54 | choronic up vs HD | |
| CCR5 | 206991_s_at |  | NM_000579 /// NM_001100168 | 2.18E-05 | 3.07 | acute up vs controller | |
| FAM153A /// FAM153B /// FAM153C | 214945_at | family with sequence similarity 153, member A /// family with sequence similarit | NM_001079527 /// NM_001079529 /// NM_173663 | 0.0218 | 1.90 | HCV high up vs HD | |
| FAM153A /// FAM153B /// FAM153C /// LOC100507387 /// LOC100507427 | 214945_at |  | NM_001079527 /// NM_001079529 /// NM_001265615 /// NM_173663 /// NR_038353 /// NR_03840 | 0.0079 | 0.49 | acute down vs HD | |
| FAM153A /// FAM153B /// FAM153C /// LOC100507387 /// LOC100507427 | 214945_at |  | NM_001079527 /// NM_001079529 /// NM_001265615 /// NM_173663 /// NR_038353 /// NR_03840 | 0.0072 | 0.49 | choronic down vs HD | |
| IFNG | 210354_at | interferon, gamma | NM_000619 | 0.0362 | 2.01 | HCV low up vs HD | |
| IFNG | 210354_at |  | NM_000619 | 0.0038 | 2.83 | acute up vs HD | |
| IGJ | 212592_at | immunoglobulin J polypeptide, linker protein for immunoglobulin alpha and mu pol | NM_144646 | 0.0244 | 0.30 | HCV low down vs HD | |
| IGJ | 212592_at |  | NM_144646 | 0.0396 | 2.86 | acute up vs controller | |
| IGK@ /// IGKC | 221651_x_at | immunoglobulin kappa locus /// immunoglobulin kappa constant | --- | 0.0408 | 0.42 | HCV low down vs HD | |
| IGK@ /// IGKC | 221671_x_at |  | --- | 0.0422 | 0.41 | HCV low down vs HD | |
| IGK@ /// IGKC /// LOC100294406 | 221651_x_at |  | XR_109956 /// XR_109957 /// XR_132705 | 0.0085 | 3.85 | acute up vs controller | |
| IGK@ /// IGKC /// LOC100294406 | 221651_x_at |  | XR_109956 /// XR_109957 /// XR_132705 | 0.0265 | 3.00 | choronic up vs controller | |
| IGK@ /// IGKC /// LOC100294406 | 221671_x_at |  | XR_109956 /// XR_109957 /// XR_132705 | 0.0092 | 3.78 | acute up vs controller | |
| IGK@ /// IGKC /// LOC100294406 | 221671_x_at |  | XR_109956 /// XR_109957 /// XR_132705 | 0.0307 | 2.90 | choronic up vs controller | |
| KLF11 | 218486_at | Kruppel-like factor 11 | NM_001177716 /// NM_001177718 /// NM_003597 | 0.0202 | 0.63 | HCV high down vs HD | |
| KLF11 | 218486_at |  | NM_001177716 /// NM_001177718 /// NM_003597 | 0.0002 | 0.38 | acute down vs HD | |
| KLF11 | 218486_at |  | NM_001177716 /// NM_001177718 /// NM_003597 | 0.0016 | 0.46 | choronic down vs HD | |
| KLRB1 | 214470_at | killer cell lectin-like receptor subfamily B, member 1 | NM_002258 | 0.0223 | 0.36 | HCV high down vs HD | |
| KLRB1 | 214470_at |  | NM_002258 | 0.0002 | 0.33 | choronic down vs HD | |
| LDLRAP1 | 221790_s_at | low density lipoprotein receptor adaptor protein 1 | NM_015627 | 0.0240 | 1.68 | HCV high up vs HD | |
| LDLRAP1 | 221790_s_at |  | NM_015627 | 0.0098 | 0.45 | acute down vs HD | |
| LEF1 | 221558_s_at | lymphoid enhancer-binding factor 1 | NM_001130713 /// NM_001130714 /// NM_001166119 /// NM_016269 | 0.0261 | 1.81 | HCV high up vs HD | |
| LEF1 | 221558_s_at |  | NM_001130713 /// NM_001130714 /// NM_001166119 /// NM_016269 | 0.0045 | 0.48 | acute down vs HD | |
| LEF1 | 221558_s_at |  | NM_001130713 /// NM_001130714 /// NM_001166119 /// NM_016269 | 0.0066 | 0.50 | choronic down vs HD | |
| LYZ | 213975_s_at | lysozyme | NM_000239 | 0.0390 | 2.24 | HCV high up vs HD | |
| LYZ | 213975_s_at |  | NM_000239 | 0.0382 | 1.95 | HCV up vs HD | |
| LYZ | 213975_s_at |  | NM_000239 | 0.0131 | 0.33 | acute down vs HD | |
| LYZ | 213975_s_at |  | NM_000239 | 0.0362 | 0.32 | acute down vs choronic | |
| MAL | 204777_s_at | mal, T-cell differentiation protein | NM_002371 /// NM_022438 /// NM_022439 /// NM_022440 | 0.0225 | 2.01 | HCV high up vs HD | |
| MAL | 204777_s_at |  | NM_002371 /// NM_022438 /// NM_022439 /// NM_022440 | 0.0120 | 0.37 | acute down vs HD | |
| MEST | 202016_at | mesoderm specific transcript homolog (mouse) | NM_002402 /// NM_177524 /// NM_177525 | 0.0340 | 1.76 | HCV high up vs HD | |
| MEST | 202016_at |  | NM_001253900 /// NM_001253901 /// NM_001253902 /// NM_002402 /// NM_177524 /// NM_17752 | 3.06E-05 | 0.29 | acute down vs HD | |
| MEST | 202016_at |  | NM_001253900 /// NM_001253901 /// NM_001253902 /// NM_002402 /// NM_177524 /// NM_17752 | 4.75E-05 | 0.30 | choronic down vs HD | |
| MEST | 202016_at |  | NM_001253900 /// NM_001253901 /// NM_001253902 /// NM_002402 /// NM_177524 /// NM_17752 | 0.0050 | 0.49 | controller down vs HD | |
| MYBL1 | 213906_at | v-myb myeloblastosis viral oncogene homolog (avian)-like 1 | NM_001080416 /// NM_001144755 | 0.0272 | 0.55 | HCV low down vs HD | |
| MYBL1 | 213906_at |  | NM_001080416 /// NM_001144755 | 0.0066 | 0.39 | acute down vs HD | |
| MYBL1 | 213906_at |  | NM_001080416 /// NM_001144755 | 0.0057 | 0.39 | acute down vs controller | |
| S100A8 | 202917_s_at | S100 calcium binding protein A8 | NM_002964 | 0.0442 | 2.67 | HCV low up vs HD | |
| S100A8 | 202917_s_at |  | NM_002964 | 0.0378 | 3.28 | HCV up vs HD | |
| S100A8 | 202917_s_at |  | NM_002964 | 0.0145 | 0.19 | acute down vs HD | |
| S100A8 | 202917_s_at |  | NM_002964 | 0.0468 | 0.21 | acute down vs choronic | |
| S100A9 | 203535_at | S100 calcium binding protein A9 | NM_002965 | 0.0296 | 2.49 | HCV low up vs HD | |
| S100A9 | 203535_at |  | NM_002965 | 0.0255 | 2.79 | HCV up vs HD | |
| S100A9 | 203535_at |  | NM_002965 | 0.0595 | 0.46 | acute down vs choronic | |
| SCML1 | 218793_s_at | sex comb on midleg-like 1 (Drosophila) | NM_001037535 /// NM_001037536 /// NM_001037540 /// NM_006746 | 0.0313 | 1.74 | HCV high up vs HD | |
| SCML1 | 218793_s_at |  | NM_001037535 /// NM_001037536 /// NM_001037540 /// NM_006746 | 0.0001 | 0.34 | acute down vs HD | |
| SCML1 | 218793_s_at |  | NM_001037535 /// NM_001037536 /// NM_001037540 /// NM_006746 | 0.0001 | 0.33 | choronic down vs HD | |
| SPATS2L | 222154_s_at | spermatogenesis associated, serine-rich 2-like | NM_001100422 /// NM_001100423 /// NM_001100424 /// NM_015535 | 0.0030 | 0.44 | HCV high down vs HD | |
| SPATS2L | 222154_s_at |  | NM_001100422 /// NM_001100423 /// NM_001100424 /// NM_015535 | 0.0017 | 2.37 | acute up vs HD | |
| SPATS2L | 222154_s_at |  | NM_001100422 /// NM_001100423 /// NM_001100424 /// NM_015535 | 0.0011 | 2.50 | choronic up vs HD | |
| SPATS2L | 222154_s_at |  | NM_001100422 /// NM_001100423 /// NM_001100424 /// NM_015535 | 0.0014 | 2.43 | acute up vs controller | |
| SPATS2L | 222154_s_at |  | NM_001100422 /// NM_001100423 /// NM_001100424 /// NM_015535 | 0.0009 | 2.56 | choronic up vs controller | |
